# A Review of Indoor Navigation Methods for Autonomous Mobile Robots: From Path Planning to Large Model-Based Decision-Making

**DOI:** 10.3390/s26216724

**Published:** 2026-10-23

**Authors:** Xiang Cheng, Shuting Wang, Yiming Hu, Yuanlong Xie

**Affiliations:** School of Mechanical Science and Engineering, Huazhong University of Science and Technology, Wuhan 430074, China; d202580353@hust.edu.cn (X.C.); wangst@hust.edu.cn (S.W.); huyiming@hust.edu.cn (Y.H.)

**Keywords:** indoor navigation, autonomous mobile robots, path planning, reinforcement learning, foundation models, vision–language navigation

## Abstract

Indoor navigation is fundamental to autonomous mobile robots (AMRs), yet the relationships between explicit planning, learned policies, and large model-based decision-making remain difficult to assess across heterogeneous experiments. This review establishes a unified taxonomy of path planning-based, learning-based, and large model-based navigation according to their dominant decision-making mechanisms, while recognizing hybrid systems. We summarize the foundations of simultaneous localization and mapping (SLAM), deep and reinforcement learning, and large models, then examine representative methods through their navigation tasks, decision roles, and validation settings. To move beyond qualitative comparisons, we synthesize reported quantitative evidence on navigation success, computational cost, online latency, dynamic adaptability, physical validation, and safety, explicitly distinguishing task protocols and measurement boundaries. We also compare common simulation platforms, datasets, and physical-robot platforms, examining how sensor noise, moving obstacles, and execution uncertainty affect sim-to-real transfer. The analysis shows that explicit planning provides interpretable, constraint-aware motion generation, learned policies support experience-driven adaptation, and large models enable semantic instruction understanding and high-level task reasoning. However, no paradigm alone resolves the challenges of generalization, embodied grounding, inference delay, and reliable physical deployment. Rather than ranking methods across incomparable evaluations, we identify complementary capabilities and the value of hybrid architectures that couple semantic reasoning with constraint-checked low-level execution and feedback. Future work should address lifelong adaptation, embodied memory and world models, efficient inference, and measurable safety and failure recovery.

## 1. Introduction

Autonomous mobile robots (AMRs) are becoming an essential component of modern indoor environments, ranging from warehouses and manufacturing facilities to hospitals and other service-oriented spaces. Driven by the rapid advancement of intelligent manufacturing, smart logistics, and human-centered service robotics, AMRs are increasingly expected to perform a broad spectrum of tasks, such as material transportation, inventory inspection, and service assistance, with minimal human intervention [1,2,3]. Fulfilling these expectations requires more than task-specific functionality; robots must be able to move autonomously and reliably through cluttered, confined, and highly dynamic environments while continuously adapting their actions to surrounding changes [4]. Indoor navigation therefore constitutes a fundamental capability that connects environmental perception with autonomous decision-making and physical interaction. More importantly, as robots evolve from preprogrammed task executors toward adaptive embodied agents, navigation is no longer merely a means of reaching a predefined destination, but a key mechanism through which perception, reasoning, and action are coupled within the physical world [5]. In this sense, advances in navigation capability are central to the broader transition of AMRs from conventional automation toward embodied intelligence.

**Figure 1:**
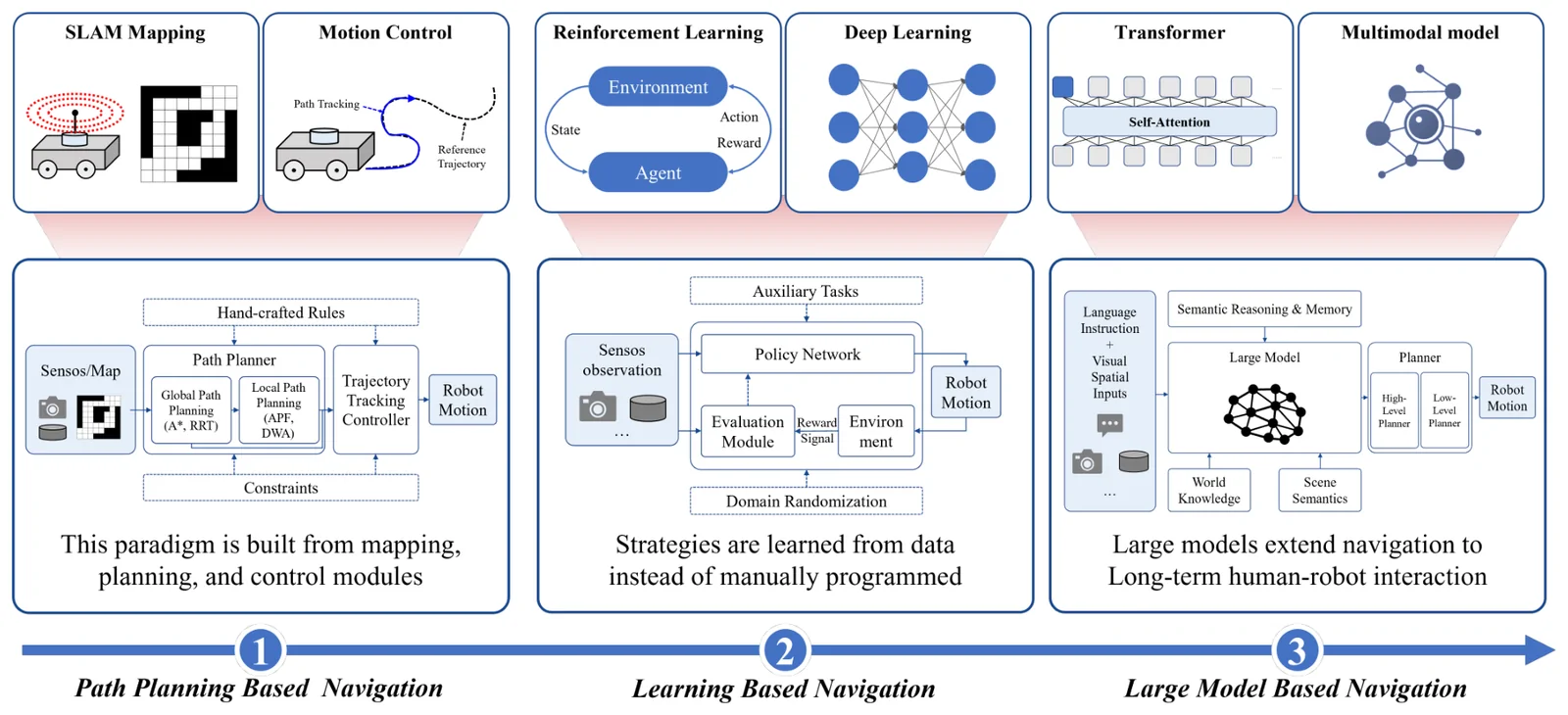
Three major technological paradigms. The general data sources required by each paradigm are indicated in the input boxes of Paradigms 2 and 3. The ellipses in the figures represent additional input designs that will be provided for the corresponding paradigms to optimize the methods.

Over the past decades, indoor navigation has undergone a progressive shift across three major technological paradigms, as illustrated in Figure 1. The early paradigm is characterized by a model-based and modular navigation architecture, in which environmental representation, path planning, and motion control are explicitly designed and executed as interconnected components. Such systems typically rely on structured representations, such as occupancy grid or topological maps, and employ explicit planning and control algorithms to generate collision-free motions toward a specified goal [6]. By decomposing navigation into well-defined modules governed by hand-crafted models and constraints, these approaches offer transparent decision processes and dependable performance in relatively structured and predictable environments. Nevertheless, their effectiveness depends heavily on the accuracy and consistency of environmental models, while substantial manual effort is often required for algorithm design and scenario-specific parameter tuning. As environments become increasingly dynamic and less predictable, discrepancies between the modeled and actual surroundings can accumulate, thereby degrading planning reliability and limiting the ability of these systems to adapt to previously unseen conditions.

To reduce the dependence on hand-crafted rules, learning-based navigation has attracted increasing attention [7]. By using data-driven techniques, AMRs can learn and summarize navigation through trial and error in the simulation environment or based on expert experience. These methods enable robots to map high-dimensional sensory inputs to navigation decisions. Learning-based approaches have shown promise in social navigation [8], visual navigation [9], and vision–language navigation [10]. Nevertheless, they also introduce new challenges. Their decision processes are often difficult to interpret, their performance depends heavily on the quality of training data, and their generalization to unseen environments remains limited. In particular, the sim-to-real gap continues to hinder the reliable deployment of learned navigation policies in physical indoor environments.

Recently, the rapid progress of large models has provided new opportunities for generalized navigation [5]. Large language models (LLMs), vision–language models (VLMs), and multimodal large models demonstrate powerful capabilities in natural language understanding, visual grounding, commonsense reasoning, task decomposition, and cross-domain generalization [11,12,13]. These capabilities make it possible to integrate high-level semantic reasoning with low-level robotic perception and control. Unlike conventional learning-based navigation models that usually learn task-specific policies from limited datasets, large model-based decision-making can exploit broad prior knowledge acquired during large-scale pretraining [14,15]. As a result, they have demonstrated great potential in high-dimensional and interactive navigation tasks such as instruction-following and embodied responses. The classification of several representative methods is enumerated in Table 1. Table 1 offers a comprehensive overview of the tasks and inputs, decision-making mechanisms, verification methods, and primary limitations of all the methods.

Existing reviews have examined various branches of autonomous navigation, including path planning [6], learning-based navigation [7], and large model-based approaches [36]. However, these reviews often emphasize methodological differences rather than the underlying evolution of navigation intelligence. To address this limitation, this review establishes a unified taxonomy based on the dominant decision-making mechanisms through which navigation behaviors are generated. Specifically, path planning-based methods derive navigation decisions from explicit environmental models and predefined optimization rules. In contrast, learning-based methods acquire decision policies through data-driven training and environmental interaction. Large model-based methods further incorporate pretrained knowledge and semantic reasoning to interpret abstract instructions and generate context-aware navigation decisions. Beyond distinguishing these paradigms by their technical characteristics, the proposed taxonomy reveals their intrinsic relationships from two complementary perspectives. With respect to environmental adaptability, navigation evolves from model-dependent replanning toward experience-driven policy adaptation and knowledge-guided semantic reasoning. In the context of robot intelligence development, the predominant source of navigation decisions has undergone a transition from handcrafted rules to learned representations and, ultimately, to higher-level cognitive reasoning. It is imperative to acknowledge that the classification criteria employed in this review are not absolute; rather, they are divided based on the core decision-making mechanism of the methods. Certain methodologies are characterized by a hierarchical or modular design, incorporating an array of approaches. This is a common practice aimed at enhancing the interpretability and robustness of the method. This approach has been employed in numerous studies. This observation is further reinforced by the data presented in Table 1. The primary contribution of this taxonomy is the provision of a unified analytical perspective for understanding the evolution, limitations, and functional complementarity of navigation paradigms, rather than merely categorizing existing algorithms.

This review provides a structured overview of indoor navigation methods for autonomous mobile robots, with a particular focus on the transition from hand-drafted path planning to learning-based navigation policy and further to large model-based decision-making. The main contributions of this review are summarized as follows.

- A unified taxonomy of indoor AMR navigation methods is established from the perspective of their underlying decision-making mechanisms, encompassing path planning-based, learning-based, and large model-based navigation. Rather than merely distinguishing methodological categories, this taxonomy reveals the evolution of navigation intelligence from rule-driven execution to experience-driven adaptation and knowledge-guided reasoning, while highlighting the intrinsic relationships and complementary capabilities among the three paradigms.
- Representative classical planners are reviewed concisely through search efficiency, motion feasibility, global–local coordination, and uncertainty. Learning-based navigation is examined through outcome-oriented design and multimodal policies. The large model-based discussion analyzes LLM task decomposition, VLM semantic and spatial grounding, closed-loop decision correction, and navigation-oriented VLA architectures.
- A comparison of robustness, generalization, and real-time deployment is supported by explicit distinctions among model roles, environmental priors, action spaces, and validation settings. Manipulation results, discrete navigation benchmarks, continuous simulation, and physical indoor navigation are interpreted within their respective evidence boundaries.
- Open challenges and future directions connect lifelong adaptation, efficient inference, sim-to-real validation, embodied memory, and world models to action-grounding, embodiment transfer, and measurable failure recovery.

## 2. Background

Indoor navigation for autonomous mobile robots is built upon several closely related technical foundations, including environment representation, localization, mapping, policy learning, semantic understanding, and high-level decision-making. Although these techniques have developed along different research trajectories, they collectively determine how a robot perceives its surroundings, estimates its state, makes decisions, and adapts to dynamic environments. This section introduces three groups of fundamental techniques that support the subsequent review: simultaneous localization and mapping (SLAM), deep learning and reinforcement learning, and large models. The selection of these three technical foundations is guided by the focus of this review on navigation decision-making rather than the complete navigation pipeline. While perception, localization, motion control, and other related technologies are essential components of autonomous navigation, the present discussion is primarily concerned with the generation and execution of navigation decisions. From this perspective, SLAM provides spatial representations and localization information that support conventional planning methods. In contrast, deep learning and reinforcement learning enable robots to acquire navigation representations and decision policies through data and interaction. The employment of large models has been demonstrated to facilitate the integration of semantic understanding and knowledge-guided reasoning, thereby supporting high-level navigation decisions. Consequently, these three groups offer the fundamental technical underpinnings for comprehending the decision-making processes of the navigation paradigms examined in Section 3, Section 4 and Section 5. In the context of navigation decision-making, other significant technologies are addressed when pertinent to the subject, as opposed to being regarded as independent classification categories.

### 2.1. Simultaneous Localization and Mapping

As the Global Navigation Satellite System (GNSS) is unavailable and unreliable indoors, the AMRs must rely on onboard sensors to infer both its location and the structure of the surrounding environment [37,38,39,40]. Meanwhile, SLAM is one of the most fundamental techniques in autonomous mobile robot navigation. It enables a robot to estimate its own pose while incrementally constructing a representation of an unknown environment. Therefore, once SLAM was proposed, it was quickly adopted and prompted.

**Figure 2:**
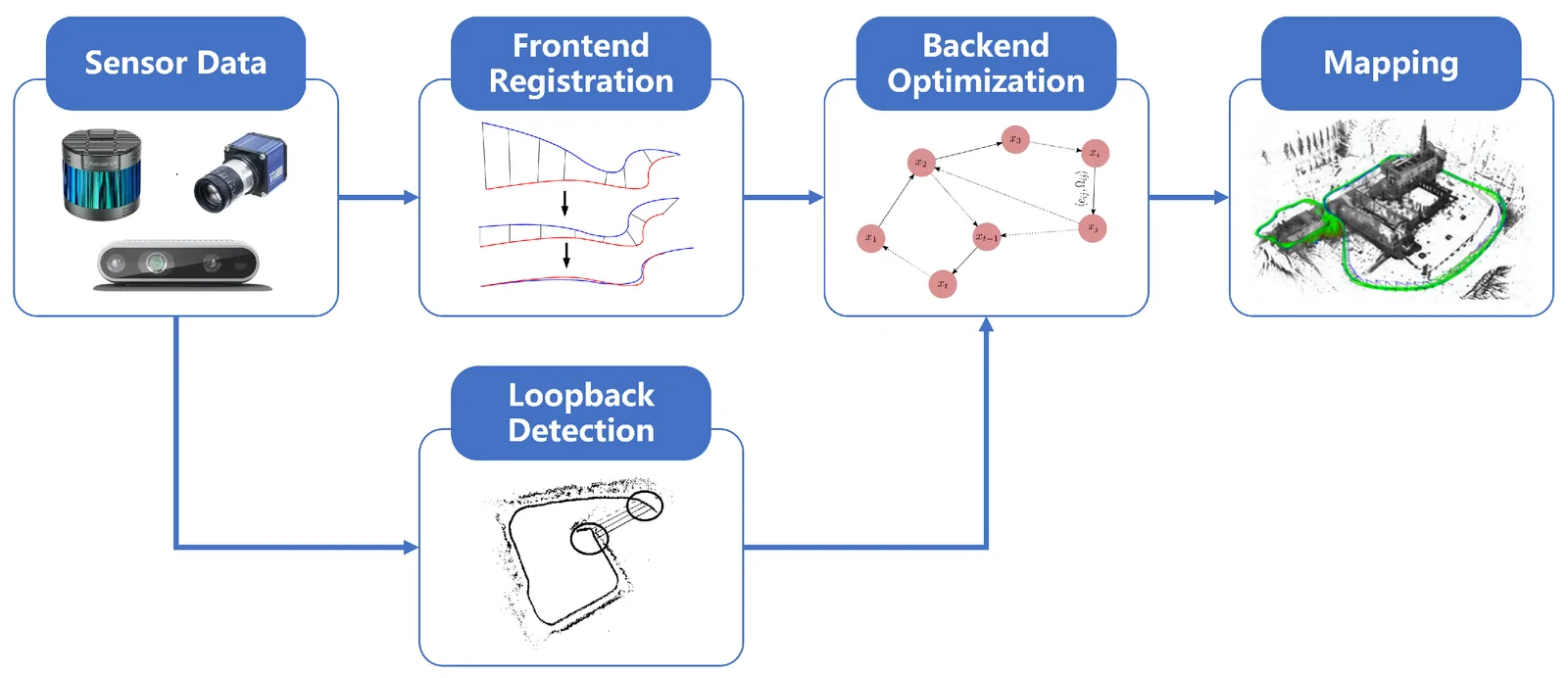
Framework of SLAM.

From the perspective of navigation, SLAM is not only a localization technique but also a mechanism for constructing environmental representations that support planning and decision-making. A typical SLAM system receives sensor observations, extracts geometric or semantic features, estimates the robot pose through data association and state estimation, and updates the map accordingly, as shown in Figure 2. The output of SLAM may take different forms, among which occupancy grid maps and topological maps are the most popular. These map representations directly influence the type of navigation strategy that can be adopted. For example, occupancy grid maps are suitable for classical path planning algorithms, while semantic or object-level maps provide richer information for task-oriented and language-guided navigation [41]. According to the dominant sensing modality and map representation, SLAM methods used in indoor navigation can be broadly categorized into LiDAR SLAM, visual SLAM, and object-level SLAM.

LiDAR SLAM relies on laser range measurements to estimate robot motion and construct geometric maps. It is widely used in industrial indoor environments because LiDAR sensors provide accurate distance measurements and are relatively robust to illumination changes. In structured scenes such as warehouses and corridors, LiDAR SLAM can generate reliable occupancy maps for localization and path planning. Its major advantage lies in geometric accuracy and operational stability [42]. However, LiDAR-based representations are often limited in semantic expressiveness [43]. A purely geometric map may indicate the presence of obstacles, walls, or free space, but it cannot directly explain what objects are present, what functions they serve, or how they relate to a navigation task. Therefore, LiDAR SLAM is highly effective for metric localization and collision-free navigation, but less sufficient for semantic reasoning or human-level instruction understanding.

**Figure 3:**
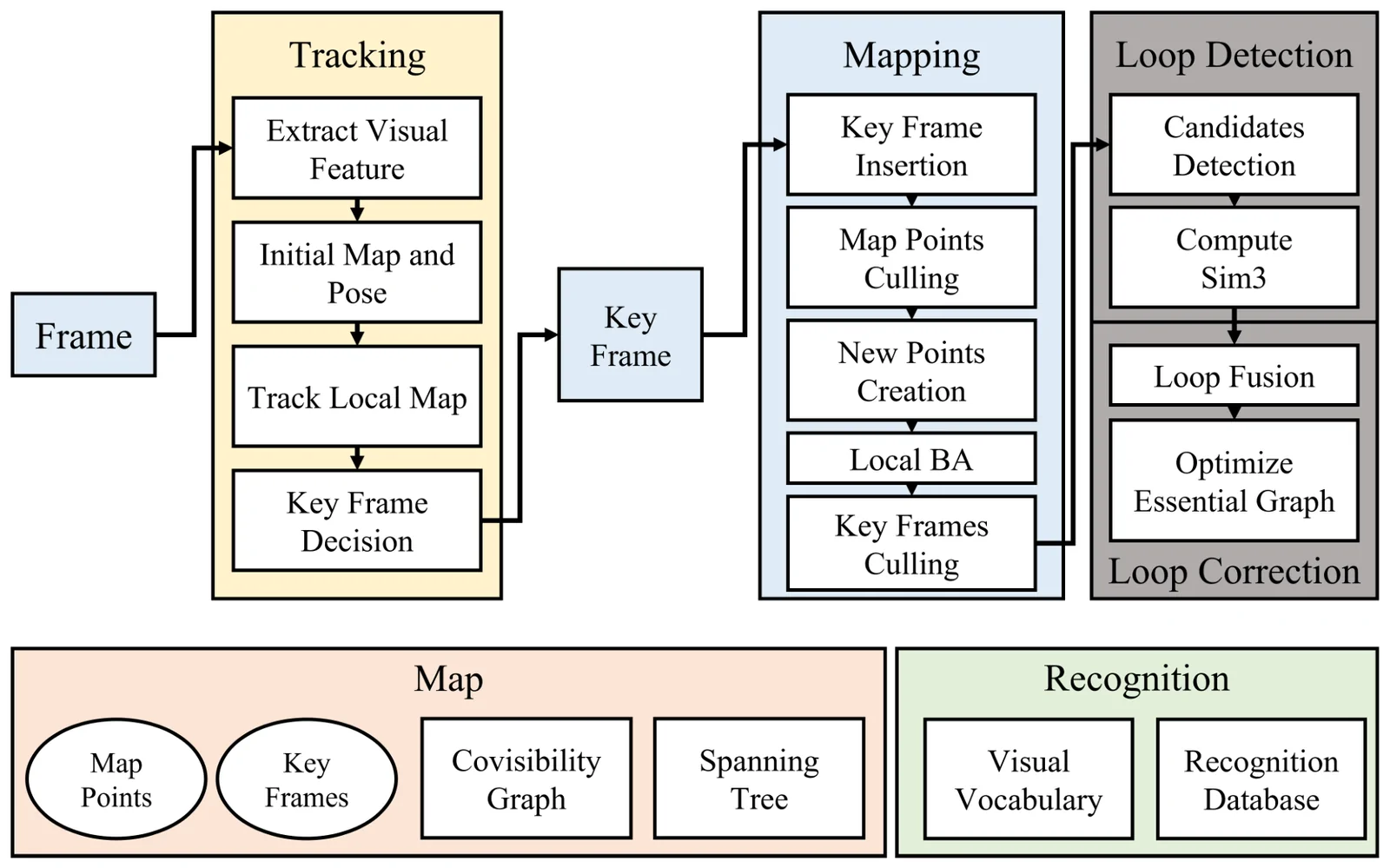
Framework of ORBSLAM.

Visual SLAM estimates camera motion and reconstructs the environment from visual observations. Compared with LiDAR SLAM, visual SLAM can capture appearance information, texture, and sometimes semantic cues, making it suitable for scenarios where spatial structures and visual features are required [44]. Among visual SLAM methods, ORB-SLAM is undoubtedly the most famous. ORB-SLAM is a real-time, feature-based SLAM system distinguished by using the same ORB features for tracking, mapping, relocalization, and loop closure, enabling efficient and robust operation [45]. Its architecture contains three parallel threads: tracking estimates camera poses and selects keyframes, local mapping manages map points and performs local bundle adjustment, and loop closing detects revisited places and corrects accumulated drift, supported by DBoW2-based place recognition and covisibility graphs, as shown in Figure 3. The original monocular system suffered from unobservable scale and difficult initialization. ORB-SLAM2 addressed these limitations by supporting stereo and RGB-D cameras with metric scale and simpler initialization [46]. ORB-SLAM3 further incorporated tightly coupled visual–inertial estimation, improved place recognition, fisheye cameras, and the Atlas multi-map framework, substantially enhancing robustness, accuracy, and long-term map reuse [47]. The strength of visual SLAM lies in its ability to provide rich perceptual information using lightweight and inexpensive sensors [48,49]. However, visual SLAM is sensitive to illumination variation, motion blur, weak texture, occlusion, and dynamic objects. In crowded or visually complex indoor environments, feature tracking and data association may become unstable. As a result, visual SLAM often requires additional mechanisms such as visual–inertial fusion, dynamic object filtering [50,51], loop closure detection [52], or semantic segmentation [53] to improve long-term robustness.

**Figure 4:**
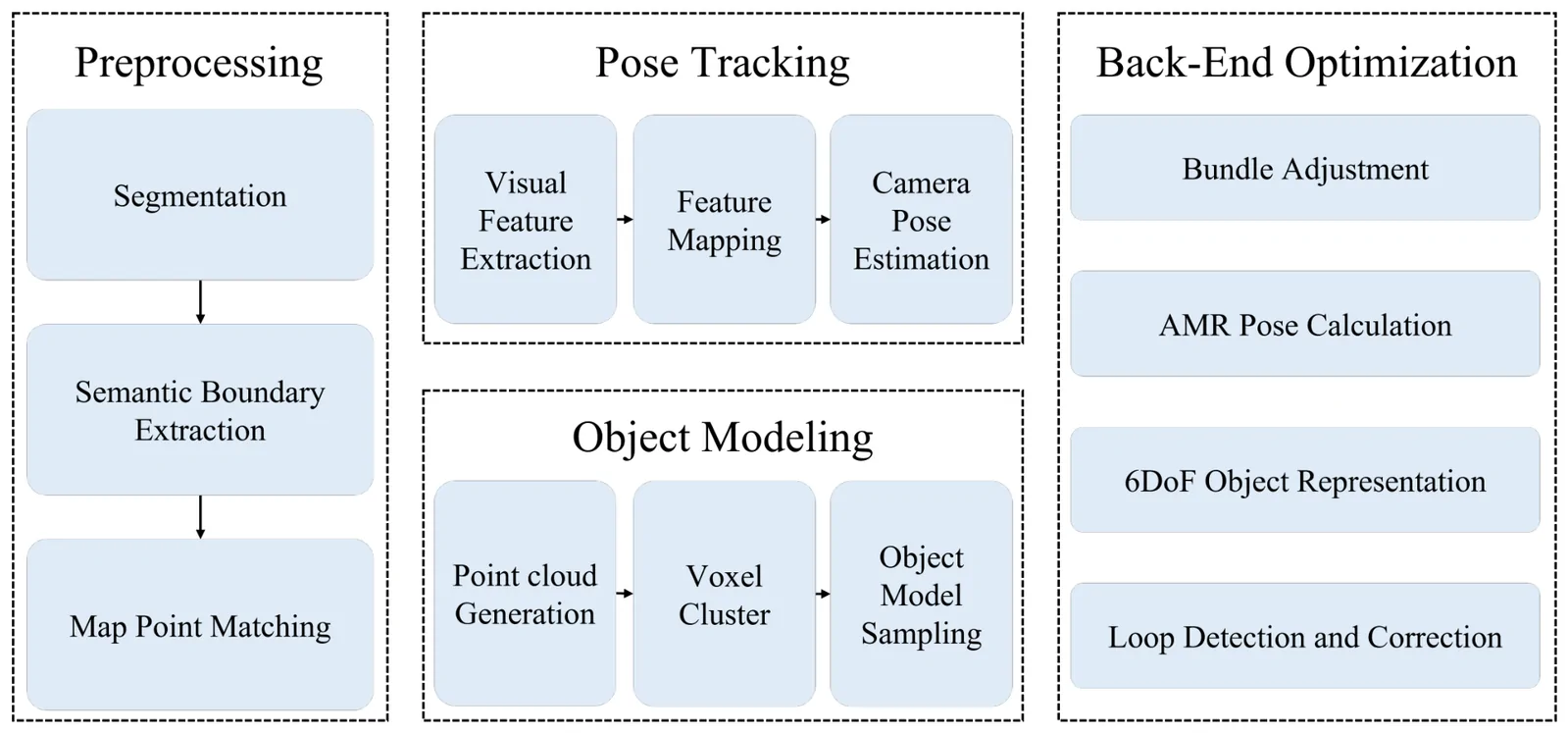
Framework of object-Level SLAM.

A typical object-level SLAM pipeline comprises four interconnected components: preprocessing, pose tracking, object modeling, and back-end optimization, as illustrated in Figure 4. These components jointly support the detection and representation of semantic objects, the estimation of camera or robot poses, and the optimization of spatial relationships between the robot and surrounding objects. It further extends conventional geometric mapping by representing the environment in terms of objects, landmarks, and semantic entities [54,55,56]. Object-level SLAM attempts to identify meaningful objects and use their spatial relationships to support localization and mapping [41,57,58]. Object-level SLAM has particular advantages for indoor navigation tasks in which target locations are specified through non-coordinate representations, such as visual observations or natural language descriptions. For instance, navigation goals may be defined by object identities or their spatial relationships with surrounding entities, such as locating a specific desk or identifying a chair near a window. These tasks introduce a fundamental gap between high-level semantic planning and low-level geometric planning and motion execution. By integrating object-level semantic information with geometric map representations, object-level SLAM provides a structured spatial representation that facilitates the grounding of abstract navigation goals in physical space, thereby bridging the gap between high-level task understanding and low-level navigation execution.

In summary, SLAM provides the spatial foundation for indoor AMR navigation by jointly estimating robot poses and constructing environmental representations. LiDAR SLAM offers accurate and reliable geometric mapping but has limited semantic expressiveness, whereas visual SLAM captures richer environmental information but remains sensitive to visual conditions. Object-level SLAM further incorporates semantic entities and spatial relationships, facilitating the translation of abstract navigation goals into geometric references, although its performance depends on reliable object recognition and association. Collectively, these methods reflect the progression from geometric mapping toward semantic representation, providing essential support for both conventional path planning and high-level navigation decision-making, while robustness in dynamic environments remains a persistent challenge.

### 2.2. Deep Learning and Reinforcement Learning

The development of learning-based navigation is closely related to the progress of deep learning and reinforcement learning. Deep learning provides the ability to extract useful representations from high-dimensional sensory inputs, such as RGB images, depth images, LiDAR scans, semantic maps, audio signals, or multimodal observations [59,60]. Instead of relying entirely on hand-crafted features, deep neural networks can learn task-relevant features from data. This makes them particularly useful for visual navigation, goal-conditioned navigation, obstacle avoidance, and scene understanding in complex indoor environments.

Reinforcement learning further extends this idea by enabling robots to learn navigation policies through interaction with an environment [61,62]. In reinforcement learning, the robot is treated as an agent that selects actions according to its current state or observation and receives rewards based on task performance. Navigation objectives such as reaching a goal, avoiding collisions, reducing travel time, maintaining social distance, or minimizing energy consumption can be encoded into reward functions. However, as the state space increases, especially in complex environments, it becomes challenging for reinforcement to represent, resulting in dimension disaster. To address this issue, neural networks are often used as function approximators to represent value functions or policies, leading to the development of deep reinforcement learning (DRL). Deep reinforcement learning allows policies to be learned from high-dimensional inputs and complex state spaces. This framework has been widely explored in indoor navigation because it can, in principle, learn adaptive behaviors that are difficult to manually design, especially in dynamic or partially observable environments [63,64,65,66,67]. The key advantage of DRL-based navigation lies in its ability to model complex perception–action relationships from data. It can reduce dependence on manually designed rules, handle high-dimensional observations, and adapt to certain environmental variations through training.

### 2.3. Large Models

**Figure 5:**
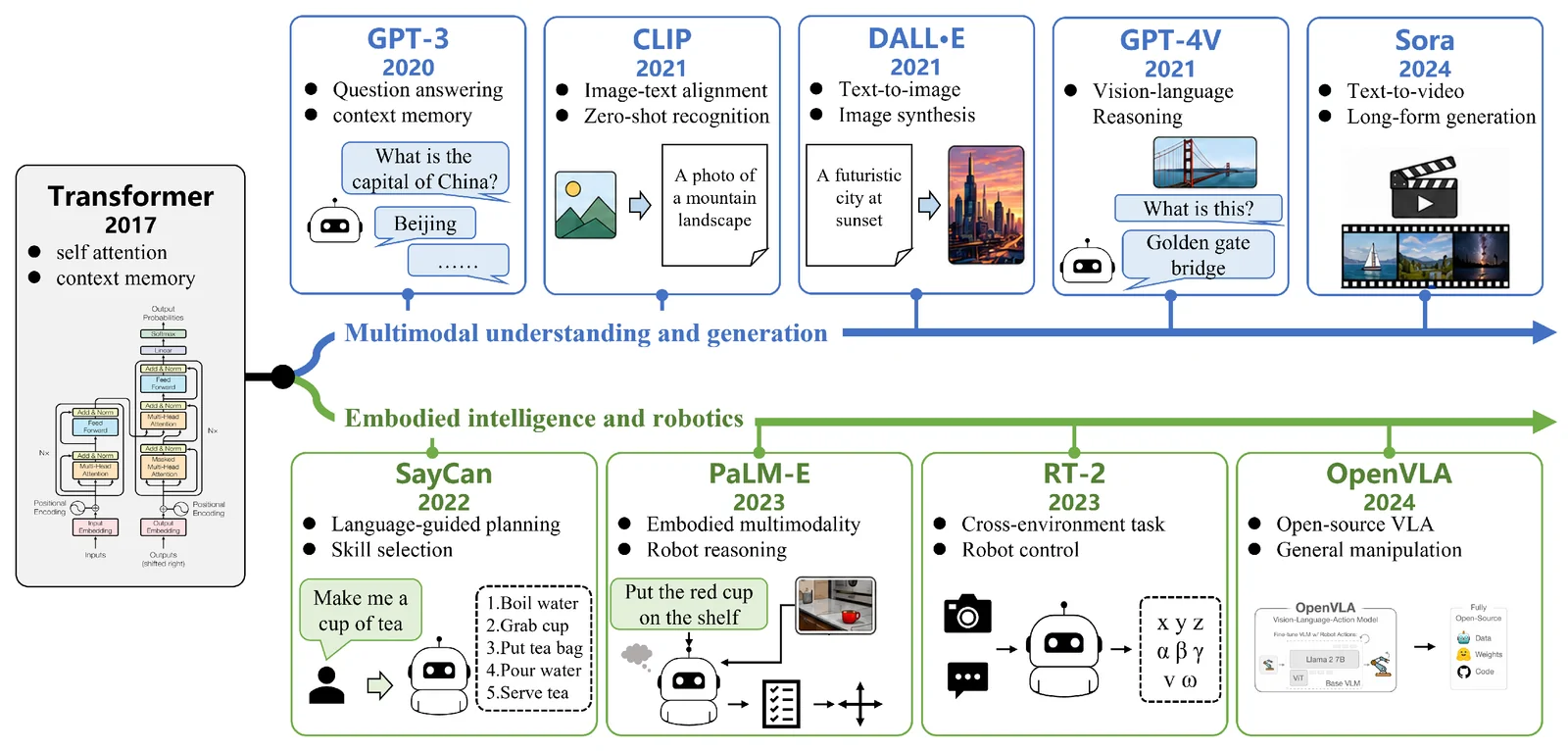
Development of large models.

Large models have recently emerged as a new technical foundation for intelligent navigation. They refer to large-scale models trained on massive and diverse datasets and capable of being adapted to a wide range of downstream tasks. Representative examples include large language models (LLMs) [11,68], vision–language models (VLMs) [69,70], multimodal large models (MLMs) [71,72,73], and vision–language–action models (VLA) [74], as shown in Figure 5. The rapid development of these models has significantly expanded the possibility of integrating semantic understanding, commonsense reasoning, and high-level task planning into robotic navigation systems.

The development of large models is closely related to the Transformer architecture [75], in which a self-attention mechanism enables models to capture long-range dependencies and process large-scale sequential or multimodal data effectively. The Transformer uses attention to mix information across tokens. For a token matrix $X\in R^{n\times d_{\mathrm{model}}}$, one self-attention head forms

(1) $$Q=XW_{Q},\;K=XW_{K},\;V=XW_{V},\;\mathrm{Attention}\left(Q,K,V\right)=\mathrm{softmax}\;\left(\frac{QK^{T}}{\sqrt{d_{k}}}\right)V,$$

where $W_{Q},W_{K}\in R^{d_{\mathrm{model}}\times d_{k}}$ and $W_{V}\in R^{d_{\mathrm{model}}\times d_{v}}$. Thus $Q,K\in R^{n\times d_{k}}$, $V\in R^{n\times d_{v}}$, and the row-wise softmax produces an n×n weighting matrix. The scale $\sqrt{d_{k}}$ controls dot-product magnitude. Multiple heads give

(2) $$\mathrm{MultiHead}\left(X\right)=\mathrm{Concat}\left(H_{1},\ldots ,H_{h}\right)W_{O},\;H_{j}=\mathrm{Attention}(XW_{Q}^{\left(j\right)},XW_{K}^{\left(j\right)},XW_{V}^{\left(j\right)}),$$

with $W_{O}\in R^{hd_{v}\times d_{\mathrm{model}}}$. In cross-attention, queries and keys/values come from different sequences and the attention matrix can be rectangular. Navigation systems can, for example, attend between instruction tokens and visual or map tokens. These equations explain information alignment, they do not establish a model’s pretraining scale, physical grounding, or safety. The same attention operation can appear in a task-trained L policy or a foundation-assisted F module.

Based on Transformer, large language models have achieved strong performance in natural language understanding, instruction following, reasoning, and text generation [12]. Vision–language models extend language models by aligning visual and textual representations. Through large-scale image-text pretraining, they can associate visual observations with semantic descriptions, object categories, spatial relations, and contextual knowledge [69]. This capability is highly relevant to indoor navigation because robots must ground abstract instructions in physical environments. Multimodal large models further integrate different types of information, such as text, images, videos, depth, audio, actions, or proprioceptive signals [72,73]. In robotic navigation, multimodal understanding is essential because no single sensor modality can fully capture the complexity of indoor environments. LiDAR provides reliable geometric structure, cameras provide visual and semantic information, language conveys human intent, and proprioceptive sensors describe the robot’s motion state. large models make it possible to process and align these heterogeneous information sources within a unified representation space, thereby supporting more flexible and context-aware navigation decisions. At the same time, a lot of high quality datasets [10,76,77,78,79,80] have been released to support the development of large models, such as Room2Room [10], Matterport3D [76], Gibson [77] and Thor [80]. These datasets provide multimodal annotations that enable large models to learn not only visual and semantic representations but also the ability to act with different language guidance.

Obviously, the emergence of large models marks a shift from experience-driven navigation toward cognition-driven navigation. This shift provides the conceptual basis for the next generation of indoor AMR navigation, where robots are expected not only to move safely and efficiently, but also to carry out tasks of a higher level, such as understanding instructions in natural language and reasoning about scenes with dynamic environments.

### 2.4. Navigation Evaluation Platforms and Datasets

The evaluation of autonomous navigation methods depends not only on algorithm design but also on the experimental environments in which these methods are tested. In indoor navigation research, simulation platforms, scene datasets, task benchmarks, and real-world robotic platforms provide complementary support for algorithm development and validation. Since their characteristics differ substantially in environmental realism, sensing conditions, task definitions, and deployment constraints, they may significantly influence the reported performance of navigation algorithms. To provide a clearer overview, the representative resources commonly used in indoor navigation research are summarized in Table 2.

As shown in Table 2, different resources emphasize different aspects of navigation evaluation. Habitat, AI2-THOR, and ProcTHOR are more suitable for large-scale embodied learning and semantic navigation studies, whereas Gazebo is particularly useful for evaluating motion planning, control, and robot-specific physical interactions. Matterport3D and Gibson provide realistic indoor scene reconstructions, while R2R and REVERIE further define task-oriented benchmarks for instruction-following and semantic grounding. In contrast, TurtleBot3 and Jackal support real-world validation under practical sensing and motion constraints.

It should be emphasized that simulation results cannot be directly regarded as equivalent to real robot performance. In simulated environments, perception is often more stable, obstacle dynamics are more regular, and robot motion is less affected by sensor noise or actuator uncertainty. By contrast, real-world deployment must cope with localization drift, sensing errors, communication latency, and unexpected environmental changes. Consequently, the same navigation method may exhibit significantly different performance in simulation and in physical experiments. For this reason, simulation platforms are more suitable for large-scale benchmarking and controlled comparisons, whereas real-world robotic platforms are essential for verifying deployment reliability and practical robustness.

## 3. Path Planning-Based Navigation Methods

Path planning-based navigation generates routes and motion commands from explicit maps, models, objectives, and constraints. Global planning establishes connectivity between the start and goal, while local planning updates executable motion using current observations. Their complementarity remains essential in hybrid navigation: a semantic model may select a destination, but geometric planning must still establish reachability and motion feasibility. This section focuses on the technical tradeoffs and recent developments that explain this continuing role.

### 3.1. Global-Path Planning

Global planners differ principally in their representation of feasible space and their allocation of search effort. Graph search, including Dijkstra and $A^{*}$, operates on discretized connectivity, sampling-based methods, including PRM and RRT, approximate connectivity in continuous configuration spaces, and metaheuristics, including PSO and ACO, to optimize candidate paths through population updates [85,86,87,88,89,90]. These formulations share a central requirement: a globally connected route must preserve obstacle clearance and provide a useful reference for downstream execution. Shortest-path optimality on a discretized graph does not establish optimality in continuous space, and collision-free connectivity alone does not ensure that a robot can follow the path under its motion constraints.

Recent developments address three connected problems. First, planning efficiency depends on informative exploration. Finer grids improve geometric resolution but enlarge the search space, unguided sampling can waste effort in irrelevant regions or fail to resolve narrow passages, and population methods may require many fitness evaluations. Lin et al. [87] improve $A^{*}$ through safety-space construction and path refinement, while learned sampling priors [91] and the evolutionary guidance of MEG-RRT* [16] concentrate sampling effort on promising regions. Improved ACO guidance and pheromone updating pursue a related objective [90]. These strategies improve the use of computation, but any claim about completeness or optimality must be checked for the particular modification and its assumptions. A learned bias may accelerate typical queries while reducing exploration of configurations poorly represented in its training data.

In addition, route quality must account for physical executability. Sharp turns, redundant nodes, and insufficient clearance can make a geometrically feasible route difficult to track. APSO combines $A^{*}$ initialization with PSO refinement [86], CAF-RRT* applies circular-arc fillets [85], and ITE-RRT* combines intermediary-tree and geometric optimization mechanisms for autonomous cars [92]. KA-IHO incorporates kinematic considerations into grid-based path optimization [93]. These examples connect search to trajectory execution, although vehicle experiments and static grid results should not be treated as interchangeable evidence of indoor AMR performance. Smoothing must also preserve clearance: shortening or rounding a path can introduce collisions unless the modified segments are checked against the robot footprint and map.

Additionally, route objectives and traversability must remain consistent with the task. Energy-aware $A^{*}$ illustrates the extension from geometric distance to operational costs [94], while ACO with rolling-window replanning combines global route guidance with adaptation during execution [95]. The important distinction is between an objective that selects a desirable route and a mechanism that maintains its validity as obstacles move. Energy, smoothness, and travel time are useful preferences, whereas collision avoidance and motion limits require feasibility checks. Changing cost weights cannot compensate for an obsolete map or a passage that has become blocked.

Consequently, global planning increasingly provides a revisable structural reference rather than a route assumed to remain valid throughout execution. Its contribution to large model-based navigation is equally concrete: semantic subgoals can be converted into map locations, screened for connectivity, and assigned feasible routes. Remaining limitations concern map error, computational scaling, objective design, and the coordination of replanning with local execution. These limitations motivate learned guidance and semantic interfaces while preserving the value of explicit route verification.

### 3.2. Local Path Planning

Local planners repeatedly convert the current state, nearby obstacles, and a global reference into short-horizon motion. APF uses attractive and repulsive potentials, DWA evaluates dynamically reachable velocity candidates, and MPC optimizes a sequence of predicted states and controls under constraints. Their principal differences concern the motion representation, prediction horizon, and computational burden. APF is inexpensive but can become trapped in local equilibria, DWA considers executable velocity choices but remains sensitive to short horizons and score weights, MPC can encode multi-step constraints but depends on model fidelity and timely optimization.

Global–local coordination addresses the conflict between immediately favorable motion and long-term progress. A locally attractive direction can lead into a concavity or away from the intended route. Path-derived virtual potential fields [96], APF-PRM coupling [97], global-path yaw information in DWA [98], and fuzzy weighting with global guidance [99] introduce route structure into local decisions. These mechanisms reduce dependence on an instantaneous goal direction, but global guidance must itself be revised when the route is blocked. Otherwise, stronger path adherence can reinforce an infeasible choice. A useful interface therefore distinguishes temporary local avoidance from a change requiring global replanning.

Context-dependent motion evaluation reduces the limitations of fixed parameters. Fuzzy DWA weights adjust competing trajectory criteria to the observed situation [99], while virtual-manipulator mechanisms modify candidate velocities using obstacle information [100]. Such adaptation can balance progress, clearance, and motion smoothness more effectively than one parameter set. Nevertheless, an adaptive score is a preference mechanism, not a proof of safety. Performance depends on sensing coverage, obstacle prediction, admissible motion generation, and the evaluation weights or adaptation rules.

Predictive planning addresses robot dynamics, uncertainty, and delayed information. Koopman-based representations and disturbance estimation improve model-based tracking [101], while residual and attention mechanisms [102] and online Gaussian-process compensation [103] address model mismatch. The evidence covers autonomous vehicles, manipulators, and mobile manipulators; these studies provide transferable control mechanisms rather than uniformly validated indoor navigation solutions. More accurate prediction also increases model and optimization complexity. Event-triggered MPC reduces repeated optimization through lighter control between events [104], and separate triggering of control optimization and model updates provides another allocation strategy [105]. The latter is demonstrated on an unmanned surface vessel, so its indoor AMR applicability requires further validation. The relevant tradeoff is whether reduced computation preserves acceptable tracking and constraint satisfaction between updates.

Prediction must also account for the environment and measurement process. Joint planning and tracking with obstacle avoidance reduces inconsistencies between independently cascaded modules [106]. ObserveNet predicts future sensory information for vehicle control [107], and uncertainty-aware active LiDAR estimation highlights the consequences of uncertain observations [108]. These approaches motivate coupling state estimation with local planning, but their sensing and platform assumptions remain part of the evidence boundary. In a dynamic indoor scene, no finite-horizon planner can ensure collision avoidance from stale or incomplete measurements alone. Update rates, prediction uncertainty, braking capability, and recovery behavior must be assessed together.

Thus, local planning is the execution interface at which global or semantic intent becomes physically constrained motion. Learned prediction, adaptive evaluation, and event-triggered optimization can improve this interface, while explicit checks and fallback behavior remain necessary. This role explains why APF, DWA, MPC, and related controllers continue to appear beneath LLM, VLM, and VLA components.

### 3.3. Summary

Path planning-based navigation derives decisions from explicit environmental representations, cost functions, feasibility constraints, and search or optimization procedures. At the global level, graph search, sampling, and metaheuristic methods determine route connectivity and path quality. At the local level, APF, DWA, and MPC translate route guidance into actions subject to nearby obstacles and robot motion constraints. Recent advancements have been made in the fields of search efficiency, motion feasibility, context-dependent evaluation, and prediction under environmental uncertainty. The central contribution of this research is the coordination of environment-level route reasoning with closed-loop local motion generation. However, the reliability of these methods is contingent upon the adequacy of maps, cost functions, motion models, and obstacle predictions. In instances where pertinent task information or environmental changes cannot be adequately encoded in these representations, algorithmic refinement alone may not adequately resolve the resulting decision uncertainty. This limitation helps explain the complementary role of navigation policies acquired through data and interaction.

## 4. Learning-Based Navigation Methods

Learning-based navigation methods refer to approaches in which an autonomous agent learns navigation decision-making from simulated environments, demonstration data, or interaction experience, rather than relying entirely on hand-crafted rules. In this paradigm, neural networks can directly or partially establish mappings from sensory observations to robot actions, thereby implicitly completing some functions traditionally handled by localization, map construction, path planning, and local control. The key logic behind this paradigm is that navigation is treated less as a sequence of manually decomposed procedures and more as a policy-learning problem constrained by inputs, outputs, rewards, training scenarios, and task objectives. Under ideal conditions, sufficient training enables the learned policy to respond to both common and exceptional navigation situations and to acquire behaviors that can be transferred to related scenarios. Learning-based methods therefore show two closely related characteristics. On the one hand, they support outcome-oriented navigation design, in which the robot learns behaviors that directly improve task success, safety, or efficiency. On the other hand, they are well suited to high-dimensional information processing and multimodal fusion, because neural networks can extract navigation-relevant features from raw or weakly processed sensor data.

### 4.1. Outcome-Oriented Navigation Design

**Figure 6:**
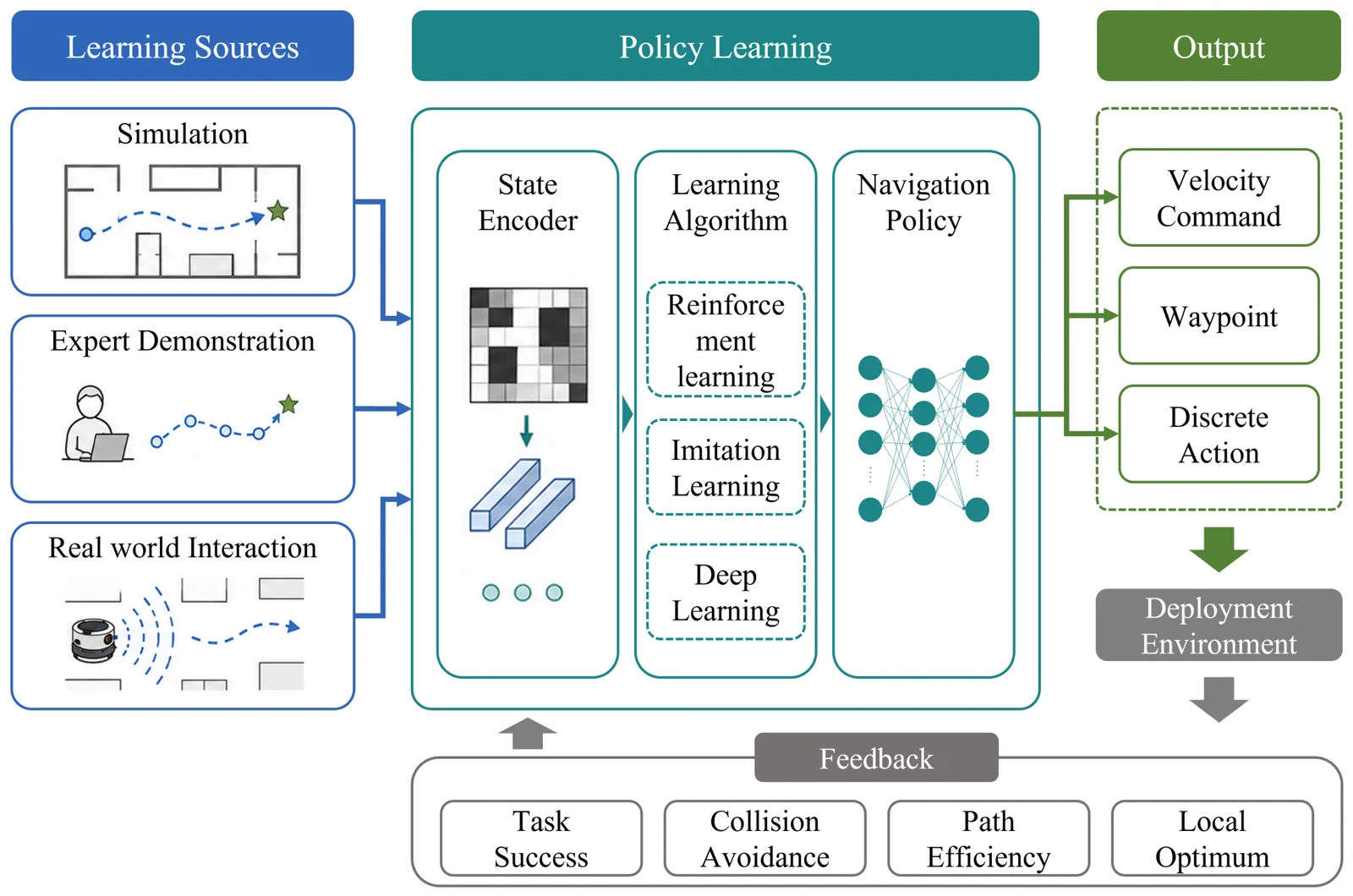
Character of learning-based navigation methods: outcome-oriented navigation design. The arrows indicate information transfer, policy learning, navigation outputs, and deployment feedback, respectively. Dashed boxes distinguish alternative learning algorithms and navigation outputs, while stars and small circles in the trajectory illustrations represent navigation goals and intermediate waypoints.

One of the most significant changes introduced by learning-based methods lies in the transition from process-oriented planning toward outcome-oriented policy optimization. Conventional path planning-based navigation usually decomposes the complete navigation task into explicitly defined processes, such as localization, path generation, obstacle avoidance, and motion control. The final behavior therefore depends on the validity of both the intermediate representations and the rules connecting these modules. Learning-based methods solve the same problem from another direction. Rather than prescribing how every intermediate navigation decision should be generated, the desired behavior is encoded through training objectives, while the agent learns which actions are more likely to produce favorable long-term outcomes, as shown in Figure 6.

This difference becomes particularly apparent when the robot encounters situations for which explicit response rules are difficult to construct. Local entrapment provides a representative example. In conventional planning, escaping a local optimum generally requires additional heuristic rules, global-path information, or auxiliary planning mechanisms. These modifications can improve individual failure cases, but the escape process must still be explicitly designed. DRL instead attempts to acquire such behavior from interaction experience. The planner does not need to analytically formulate an escape trajectory for every obstacle configuration, rather, training encourages actions that eventually recover navigation progress. Jang et al. [109], for instance, introduced sub-target generation to guide the robot around locally unfavorable regions and employ HIT-RL to utilize intermediate experience more effectively. Hu et al. [110] improved the soft actor-critic framework through a dynamic temperature coefficient together with exploration rewards and auxiliary tasks, enabling the agent to acquire both obstacle-avoidance and escape behaviors during training. In these approaches, the desired navigation response is progressively shaped through feedback instead of being fully encoded by analytical rules.

The same outcome-oriented logic is also evident in dynamic obstacle avoidance. Mobile robots operating around pedestrians or moving objects must continuously select actions under partial observations and uncertain environmental evolution. Explicit planning can incorporate obstacle motion models, but its performance becomes strongly dependent on the accuracy of these models and the validity of short-term predictions. Learning-based navigation instead optimizes the relationship between observed dynamic situations and actions that improve safety and task completion [111,112,113]. Gao et al. [114] employed an LSTM-based encoder to transform observations containing a variable number of dynamic obstacles into a fixed-dimensional representation, allowing the policy to operate in stochastic working environments. Wang et al. [115] further combined GRU-based hierarchical environment modeling, attention mechanisms, and adaptive reward functions to improve collision-avoidance decisions in complex scenes. Rather than separately prescribing how each obstacle should be handled, these approaches allow the policy to learn navigation responses from the relationship between observed interactions and their eventual consequences. Recent research therefore increasingly concerns not only the choice of reinforcement learning algorithm, but also how navigation outcomes should be represented during training. Sparse task-success signals alone may provide insufficient information for learning complex avoidance and recovery behaviors. Consequently, intermediate objectives, exploration incentives, adaptive rewards, auxiliary tasks, and structured subgoals are progressively introduced to provide more informative training feedback. This development reflects an important characteristic of outcome-oriented navigation, that although learning-based navigation reduces the need to explicitly formulate every decision rule, navigation requirements must still be translated into training objectives that can correctly shape policy behavior. The effectiveness of learning-based methods consequently depends not only on policy-network capability, but also on whether the learning signal adequately represents the intended navigation outcome.

### 4.2. Multimodal Driven Navigation

**Figure 7:**
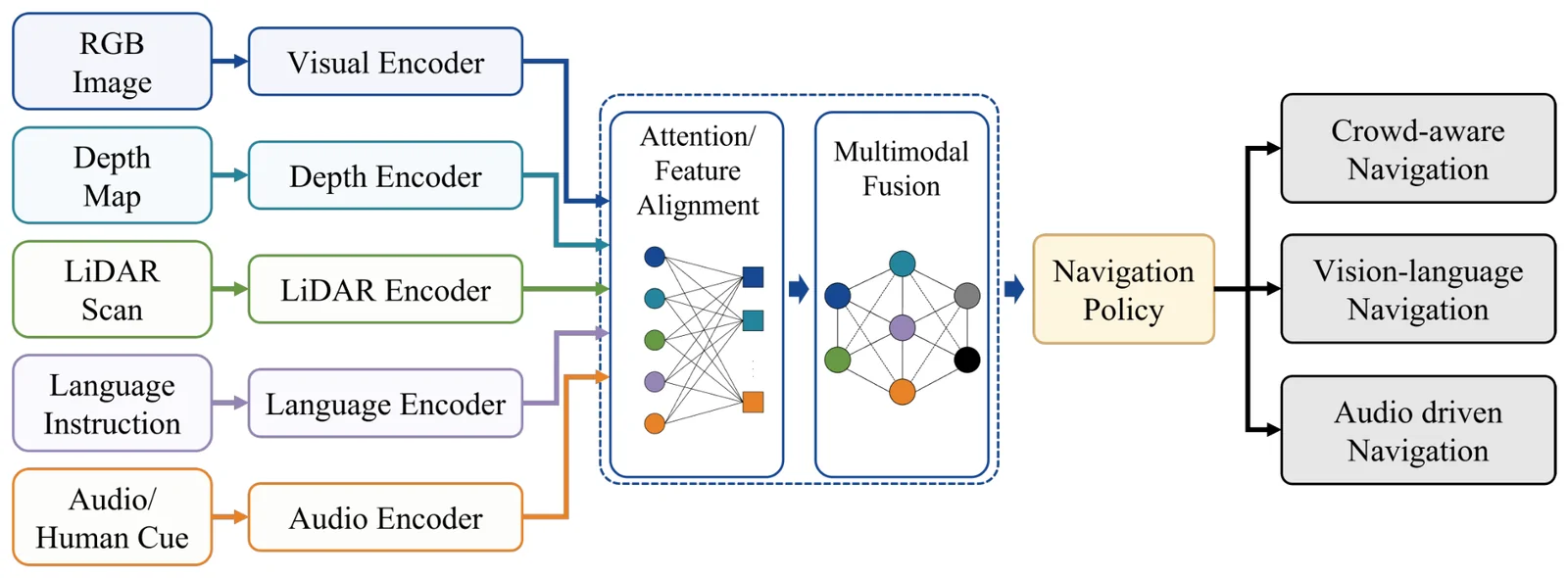
Character of learning-based navigation methods: multimodal driven navigation. The differently colored arrows and nodes represent distinct input modalities, including RGB images (blue), depth maps (cyan), LiDAR scans (green), language instructions (purple), and audio cues (orange). The dashed boundary groups the feature alignment and multimodal fusion modules, while solid arrows indicate the direction of information processing. Circular nodes represent modality-specific features and their interactions during feature fusion.

Beyond policy optimization itself, learning-based navigation methods have also become closely associated with the increasing use of high-dimensional and heterogeneous sensory information in mobile robot navigation. Traditional planners generally require environmental observations to be converted into explicitly defined quantities before they can be used by the planning algorithm. In contrast, learning policies can combine feature extraction and action evaluation within a learned decision framework, as shown in Figure 7, making it possible to derive navigation behavior from richer observations such as LiDAR measurements, visual information, temporal motion histories, and goal-related features [116,117,118]. The importance of this capability is not simply that more sensor inputs can be introduced; rather, learned representations can capture relationships among these inputs that are difficult to encode through manually designed state variables [19,119].

Crowd navigation provides a representative setting in which this capability becomes essential. In pedestrian-rich environments, collision avoidance alone is insufficient because robot decisions also depend on the temporal evolution and mutual interactions of surrounding agents [120]. Instantaneous geometric positions cannot completely describe whether a pedestrian is approaching, yielding, crossing, or moving cooperatively. Learning-based methods, such as DRL, enable navigation problems to be formulated as sequential interaction rather than static geometric avoidance. Chen et al. [121] introduced Collision Avoidance with Deep Reinforcement Learning (CADRL), representing multi-agent collision avoidance as a sequential decision-making problem and learning cooperative avoidance behavior through DRL. This formulation established a foundation on which subsequent studies increasingly enriched the information supplied to the navigation policy. Researchers have subsequently put considerable attention on representing the temporal and relational structure of crowd interactions. Liu et al. [122] introduce recurrent modeling to capture temporal dependencies in pedestrian trajectories, allowing the robot to make decisions according to motion evolution rather than relying exclusively on instantaneous observations. Li et al. [123] further represent robot–crowd interactions through directed spatio-temporal graphs, enabling the learning policy to explicitly capture relationships among multiple agents in dense environments. Other socially aware formulations introduce danger regions, adaptive warning zones, or behavior-oriented reward terms to encourage larger safety margins and navigation behavior that better reflects human motion patterns [8,124]. These studies reveal a broader tendency in multimodal navigation that improvements increasingly arise from how environmental information is temporally encoded, relationally organized, and supplied to the decision policy, rather than simply from increasing network depth or changing the underlying reinforcement learning algorithm.

Goal-conditioned visual navigation further extends this principle from dynamic interaction to perceptually specified navigation objectives. Unlike coordinate-based navigation, the destination may be represented by an image or another perceptual description, requiring the robot to determine how the current observation relates to the desired target before selecting an action. Such tasks are difficult to formulate through conventional geometric planning when explicit target coordinates are unavailable. Learning-based formulations can instead embed goal information together with current environmental observations into a shared policy-learning process [125,126]. Rao et al. [9] employed inverse dynamics modeling, multi-goal co-learning, and self-supervised auxiliary objectives to mitigate sparse rewards and improve navigation across multiple goals and unseen scenes. The significance of these mechanisms lies in their ability to enrich the internal representation used by the policy, thereby allowing reinforcement learning to operate on more informative perceptual features rather than on manually specified geometric states alone.

Taken together, multimodal-driven navigation has evolved from directly mapping relatively simple sensor states to actions toward constructing richer representations of temporal evolution, agent interaction, perceptual goals, and contextual information. This progression substantially enlarges the range of navigation problems that can be addressed through learned policies, particularly when appropriate actions depend on information that cannot be sufficiently represented by static maps or predefined geometric variables. At the same time, incorporating increasingly complex observations also strengthens the dependence of the learned policy on training data and representation quality. A policy may perform effectively within scenarios similar to those encountered during training while exhibiting degraded behavior under unfamiliar sensor distributions or environmental structures.

Beyond deep reinforcement learning, recent studies have also explored the integration of learning techniques with reactive control and visual navigation. Stavrinidis and Zacharia [20] introduced an adaptive neuro-fuzzy inference system (ANFIS) combined with conventional path planning to achieve collision-free navigation in dynamic environments. By employing adaptive controllers for goal tracking and obstacle avoidance, this approach improves navigation smoothness while reducing the complexity of fuzzy control rules. From another perspective, Al-Saedi et al. [21] proposed a visual navigation framework integrating event-camera-based depth estimation with a hybrid CNN-LSTM network, demonstrating the potential of learned visual representations for efficient navigation. These studies illustrate how learning-based techniques can enhance conventional navigation through adaptive control and improved environmental perception. Nevertheless, their performance has primarily been evaluated through simulation-based experiments, and further real-world validation is needed to establish their robustness and generalization. Such developments highlight the diversity of learning-based navigation beyond conventional reinforcement learning frameworks.

### 4.3. Summary

Learning-based navigation generates decisions through representations and policies acquired from data, demonstrations, or interaction. Outcome-oriented optimization uses rewards, intermediate goals, and auxiliary objectives to shape task completion and avoidance behavior, while multimodal learning incorporates temporal, relational, and goal-specific information into action selection [127,128]. These mechanisms are particularly useful when the relationships between observations and appropriate actions are difficult to describe through explicit rules. Their applicability must nevertheless be evaluated in relation to training objectives, observation quality, and the distribution of environments and tasks used for training and testing [129,130]. In particular, performance in familiar simulated scenes does not by itself establish robustness in unseen or physical environments. Learned policies therefore offer an additional source of navigation capability, but their contribution must be assessed together with the sensing, planning, and control components through which actions are ultimately executed.

## 5. Large Model-Based Navigation Methods

By incorporating large-scale prior knowledge into robotic decision-making, large models enable AMRs to better interpret human instructions, understand semantic scene context, and adjust navigation decisions based on multimodal observations. Compared with previous navigation paradigms, large model-based methods introduce a broader form of generalization [131]. Conventional learning-based policies are typically optimized for specific tasks or environments, whereas large models can transfer knowledge acquired during large-scale pretraining to previously unseen navigation scenarios. Such transferability is particularly valuable in real-world environments, whose spatial configurations and task conditions cannot be exhaustively modeled in advance. When encountering unfamiliar layouts or ambiguous instructions, large models can exploit linguistic and visual context to infer task intent and identify relevant navigation cues, thereby reducing the reliance on densely annotated task-specific data [23]. Beyond generalization, large model-based navigation also provides greater adaptability to dynamic and partially observed environments. Path planning-based methods generally operate on explicit spatial representations and predefined optimization objectives, making their performance sensitive to the completeness and accuracy of environmental models. In contrast, large models can interpret evolving scene information through vision–language representations and incorporate semantic context into decision-making [132]. However, their role is primarily at the level of high-level reasoning rather than low-level motion control. They can assist with subgoal generation, target-oriented exploration, and action assessment, while conventional planning and control modules remain responsible for safe trajectory generation and execution. Under incomplete or noisy observations, such semantic reasoning can further constrain the set of plausible actions, reducing the dependence of navigation decisions on rigid environmental assumptions. Another important advantage lies in the closer integration of natural language instructions with robotic execution. Indoor navigation tasks are increasingly specified through human commands rather than explicit coordinates, and these commands often contain implicit goals or context-dependent requirements [131]. Large models can interpret such abstract instructions and convert them into navigation-relevant goals or intermediate decisions, which can subsequently be executed by conventional planning and control modules. Large model-based navigation can therefore be viewed as a hybrid framework in which semantic reasoning supports high-level decision-making, while geometric planning and motion control ensure reliable physical execution.

The role of large models in navigation varies depending on the type, as shown in Table 3. An LLM, due to its strong language understanding and response capabilities, is often used in navigation to receive and analyze language instructions, and perform task reasoning and high-level decision-making, and is commonly employed in tasks that involve following instructions for navigation. Because of the presence of a large amount of multimodal information during the navigation process, such as images, a semantic map, and the AMRs’ state; these types of information cannot be fully processed appropriately by the LLM. Therefore, the VLM is often used in navigation to process multimodal information in order to physically ground language instructions. The VLA model further expands the scope of large models from a single decision-making process to an execution process. By learning skills through interaction with the environment, the VLA has pushed the role of large models to a new level.

**Figure 8:**
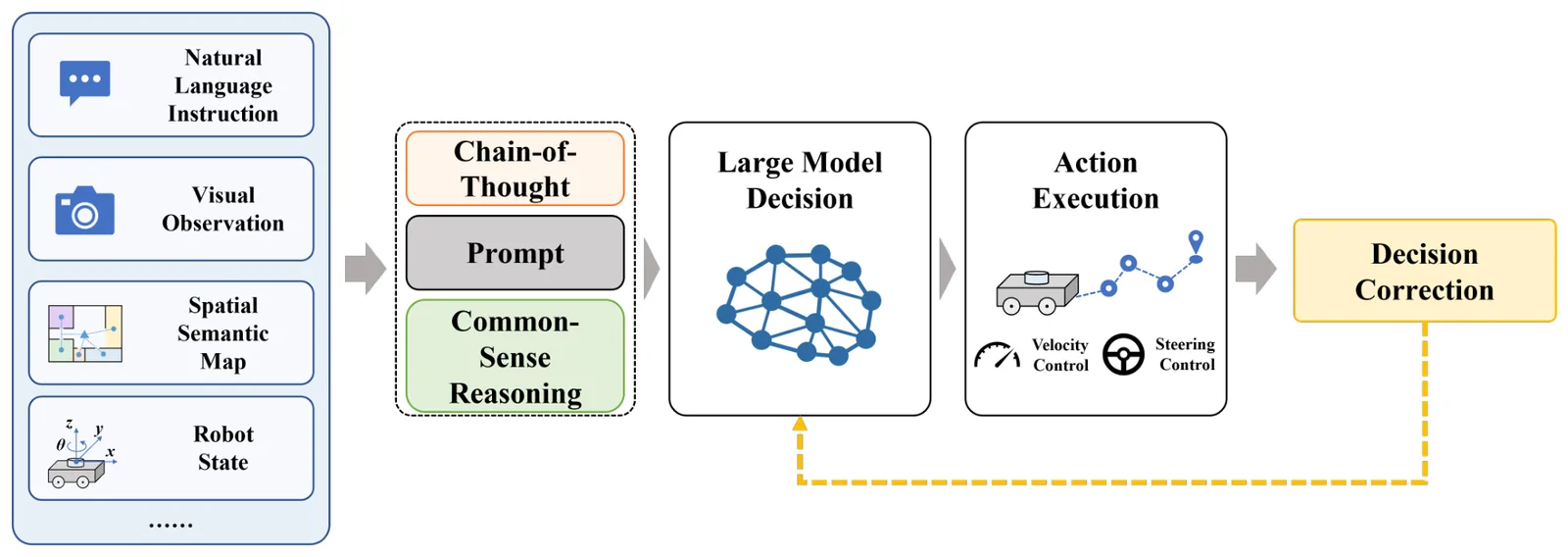
Large model-based navigation method paradigm. The gray arrows show the flow of information from the multimodal inputs to the reasoning, decision-making, action execution, and correction. The yellow dashed arrow represents the feedback loop that refines subsequent decisions.

Research on large model-based navigation has achieved substantial progress in several key technical areas, yet a number of challenges remain unresolved. As illustrated in Figure 8, existing approaches have primarily evolved along three complementary directions. First, chain-of-thought reasoning is introduced to interpret multimodal instructions, decompose complex tasks into executable subgoals, associate these subgoals with the surrounding environment, and guide action selection. Second, commonsense reasoning incorporates prior knowledge of scene organization, spatial semantics, and task relations into the decision-making process, enabling the model to better align instructions with environmental context and prioritize plausible actions. Third, closed-loop feedback and decision correction continuously compare planned behavior with actual execution, derive feedback from real-time observations, and refine subsequent decisions accordingly. Together, these mechanisms improve the robustness, adaptability, and safety of large model-based navigation in dynamic and uncertain environments.

### 5.1. Chain-of-Thought-Based Task Decomposition

**Figure 9:**
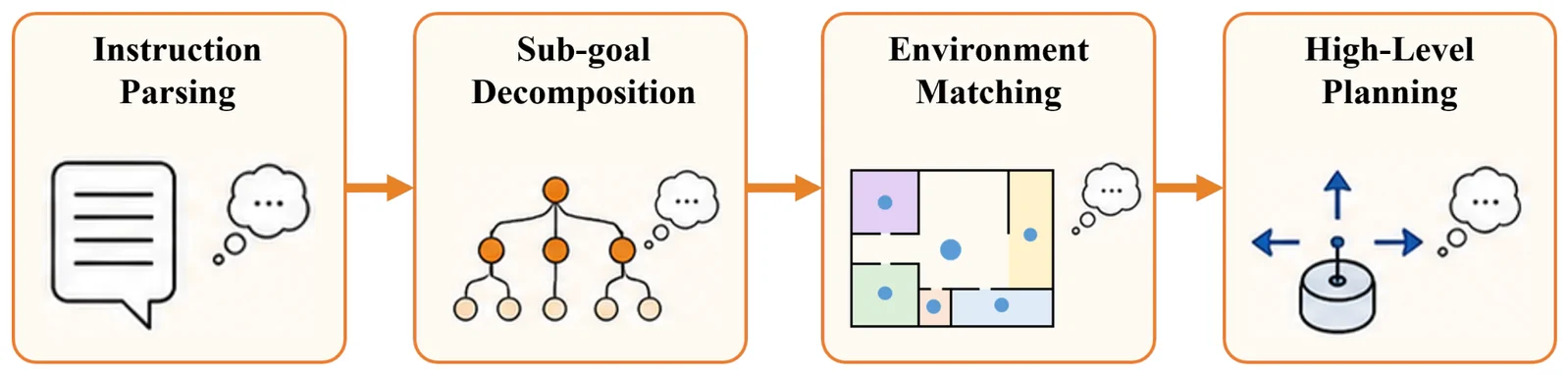
Scheme of chain-of-thought. The arrows show the order of progression from parsing instructions to decomposing subgoals, matching the environment, and high-level planning. Orange circular nodes represent hierarchical subgoals, and blue dots represent candidate spatial locations or landmarks in the environment. The colored map regions distinguish different spatial areas.

Language instructions express destinations, relations, and task dependencies that must be converted into navigation decisions. An instruction to visit an office and approach a chair next to a desk requires destination identification, landmark grounding, route selection, and completion assessment. If a large model directly generates navigation actions without an intermediate thinking process, the resulting decisions may be semantically reasonable yet physically infeasible. This limitation becomes particularly evident in zero-shot scenarios, where the robot is expected to accomplish unfamiliar tasks without task-specific training. In 2022, Wei et al. [136] introduced chain-of-thought (CoT) prompting, in which intermediate thinking steps are elicited through illustrative examples before the final prediction is produced. This thinking paradigm provides a useful foundation for decomposing complex navigation tasks with large models. Rather than generating an action immediately, CoT encourages the model to first construct an intermediate thinking sequence that links high-level instructions to executable decisions. In navigation tasks, this process often involves decomposing a long-horizon instruction into a sequence of spatially grounded subgoals. For example, the command “go to the office and find the chair beside the desk” requires the robot to first identify the destination, then recognize the relevant landmark, and subsequently determine an appropriate approach position, as shown in Figure 9. Through such hierarchical decomposition, CoT converts an abstract navigation objective into a structured sequence of intermediate decisions that can be grounded, evaluated, and executed step by step.

The effectiveness of CoT primarily stems from its ability to elicit structured multi-step thinking from large models. By incorporating task decomposition into the prompting process, CoT enables the model to analyze instruction structure and maintain logical consistency across successive decision stages [14,137]. This capability is particularly important for navigation, where local actions must remain aligned with the global task objective throughout execution. The thought chain therefore serves as an intermediate semantic layer that connects high-level task interpretation with low-level embodied actions. A representative example is NavGPT, proposed by Zhou et al. [32], which generates explicit thinking for selecting a particular direction or subgoal rather than directly producing an action command. Such intermediate thinking improves the interpretability of the navigation process and facilitates failure analysis when incorrect decisions occur. NavGPT-2 subsequently strengthened the coupling between thinking and action by integrating thinking chains with a policy network and allowing action feedback to affect subsequent decisions [33]. During training, GPT-4V is used to generate navigation trajectories, enabling the model to learn structured mappings from visual observations to actions. Compared with purely end-to-end navigation policies, this architecture preserves the thinking capability of foundation models while improving their integration with embodied control. Subsequent studies have extended CoT-based navigation from different perspectives. Kong et al. [138] introduced landmark-centered reasoning to improve the consistency of instruction generation, while Li et al. [139] incorporated scene-graph knowledge to constrain subgoal selection. Lin et al. [140] further adjusted the granularity of task decomposition according to environmental uncertainty. Collectively, these developments suggest that CoT is evolving from a general prompting technique into a more structured thinking mechanism tailored to embodied navigation.

NavGPT illustrates a prompting-based interface [32]. It receives textual descriptions of visual observations, navigation history, and candidate directions, and uses an LLM to select successive viewpoints. Its thinking can expose subgoal decomposition, landmark identification, and progress tracking. However, the original zero-shot R2R performance remains below that of trained navigation specialists. This result is instructive: broad linguistic competence can support navigation thinking without supplying the spatial representations or action experience of a specialist policy. Furthermore, converting images to text creates an information bottleneck, because omitted geometry or misidentified landmarks cannot be recovered reliably from language thinking alone. The discrete candidate-viewpoint setting also limits what can be inferred about continuous obstacle avoidance. NavGPT-2 addresses part of this gap by aligning visual observations with a frozen language model and coupling the resulting representations to a navigation policy network [33]. This changes the interface from text-only prompting toward learned visual grounding and action prediction while retaining linguistic thinking. The study reports improved navigation performance and data efficiency on its VLN benchmarks. The important distinction is that this improvement includes navigation-specific training: it should not be interpreted as evidence that an unchanged general-purpose LLM can achieve the same behavior without adaptation. Comparing NavGPT and NavGPT-2 therefore highlights a shift in the location of learning, from external observation descriptions and prompting toward visual alignment and a task-trained policy.

Maintaining a decomposition over time additionally requires memory and instruction disambiguation. MatchNav addresses these problems through CognitionMatcher for short-term memory consolidation, EnvMatcher for environment-related matching, and InstructionMatcher for reducing semantic ambiguity [132]. This places the contribution at the interpretation and memory interface, rather than at low-level trajectory execution. Its navigation results should be read within the tasks evaluated in the source. More generally, retrieving an experience can suggest how to interpret a new instruction, while current observations must still establish whether the suggested landmark or route applies. Experience matching therefore complements task decomposition without making a previously successful plan automatically valid in a changed scene.

FASTNav approaches the deployment problem through compact pretrained language models adapted to multi-point navigation [27]. It combines fine-tuning and teacher–student iteration, produces ordered navigation goals, and passes them to Nav2. The paper reports physical-robot experiments and local deployment on a Jetson Orin NX. Its task-specific adaptation can reduce the amount of map information required in each prompt, but this also ties the demonstrated behavior to the training and map context. Compact-model deployment and generalization to an unfamiliar building are distinct claims. The example further shows that a foundation-assisted decision mechanism can be retained after model compression; parameter count alone is an inadequate classification criterion.

Across these approaches, the useful intermediate representation is a subgoal whose meaning and completion can be checked. For navigation, such a representation should identify the target or landmark, the intended spatial relation, the evidence supporting it, and a termination condition. A coordinate waypoint is appropriate after metric grounding, while a semantic instruction may be necessary before the target is observed. The system must distinguish these states: an inferred destination should not be presented to the controller as a verified location. This is a design implication of the reviewed interfaces, rather than a claim that every cited method implements the same subgoal schema.

Task decomposition also balances between granularity and error propagation. Large subgoals reduce thinking calls but place greater demands on the executor; very small subgoals improve opportunities for checking progress but increase inference cost and may cause repeated replanning. In partially observed environments, committing to a complete fixed sequence is particularly fragile. A more appropriate strategy is to retain the overall task while resolving the next subgoal with the evidence currently available. Completion monitoring must then check observed progress, not merely whether the model has generated the next step. Navigation thinking should be evaluated through task completion, subgoal grounding accuracy, recovery from invalid decompositions, and the cost of repeated inference, with the contribution of the lower-level policy reported separately.

A particularly informative comparison would hold perception, candidate actions, and the executor fixed while changing the reasoning interface. Prompt-only decomposition, visually aligned thinking, and learned policy coupling could then be assessed under the same instructions and observations. Otherwise, an apparent gain from CoT may also reflect a stronger visual encoder, better candidate generation, or more navigation training. Intermediate explanations remain useful for inspection, but the review should attribute performance to tested components and verify whether the explanation remains consistent with the chosen action. This requirement connects interpretability to measurable behavior rather than treating the existence of a trace as sufficient evidence.

### 5.2. Commonsense Reasoning

**Figure 10:**
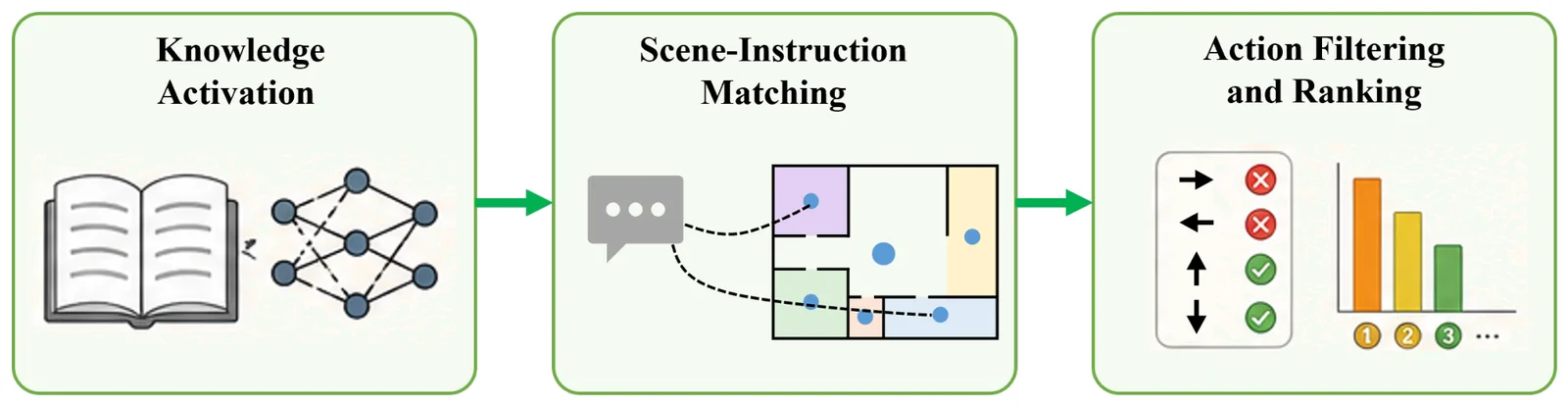
Scheme of commonsense reasoning. It illustrates the progression from knowledge activation to scene-instruction matching and action filtering. Dashed lines show the semantic connections between language instructions and candidate spatial locations, indicated by blue dots. Red crosses and green checkmarks indicate rejected and retained actions, respectively. The differently colored bars show their relative rankings.

Commonsense reasoning is most useful when a target is unobserved or an instruction leaves its destination implicit. A robot may need to locate an object outside its current field of view or interpret an instruction in which relevant spatial information is only implicitly specified. Conventional learning-based methods often infer such relationships from patterns acquired during training, but these learned correlations may generalize poorly to previously unseen environments. Commonsense reasoning addresses this limitation by incorporating prior knowledge into the navigation process [141,142]. The basic principle is to exploit general knowledge about scene organization and task relationships to infer plausible navigation decisions, as shown in Figure 10. For example, when searching for a cup, a robot may prioritize areas such as a kitchen rather than exploring the environment without guidance. Such reasoning does not replace perceptual observations; instead, it complements them when sensory information is incomplete or ambiguous. In indoor navigation, commonsense knowledge can therefore help estimate likely target locations, identify plausible actions, and assess whether a candidate plan is consistent with typical human expectations.

VLMaps makes this connection explicit by fusing pretrained visual-language features with a 3D reconstruction [143]. Language queries can then retrieve spatially localized semantic content, including relational navigation goals, while geometric representations support movement. The paper includes simulated and physical-robot experiments. Its contribution is the map interface between language and space, rather than replacing localization and motion planning with a language model. The resulting representation can reduce dependence on a fixed object vocabulary, but its usability still depends on reconstruction quality and whether the queried relation is supported by sufficiently accurate spatial evidence.

OVL-MAP develops online visual-language mapping for continuous navigation [28]. Pretrained LSeg features are associated with RGB-D geometry, while BERT/LSTM-based modules support waypoint prediction and DD-PPO supplies learned action decisions. This is a combination of pretrained semantic representation and task-trained navigation, rather than an autoregressive LLM planner. Its Habitat evaluation establishes evidence in continuous simulated environments, not physical indoor deployment. The distinction matters because online map updates may improve semantic access while still accumulating pose and feature-association errors. Open-vocabulary perception and reliable long-horizon execution must therefore be evaluated separately.

Language Frontier Guide (LFG) uses LLM knowledge in a different position: it biases exploration toward promising candidates in an unfamiliar environment [144]. Semantic observations inform a language-based search heuristic, while metric or topological planning handles movement. Unlike language-to-goal grounding for an already observed object, this mechanism helps decide where to search before the destination is known. The LLM’s prediction functions as a preference over uncertain search options. Its physical navigation experiments support the usefulness of semantic guidance in the tested environments, while the architecture preserves a separation between conjectured target likelihood and traversable space.

CoNav provides a complementary approach to spatial ambiguity [145]. A pretrained 3D-text model communicates structured spatial–semantic hypotheses to an image-text navigation agent through cross-modal belief alignment and lightweight fine-tuning. Its evaluations include embodied navigation and spatial reasoning benchmarks. The method should be understood as cross-modal guidance, rather than as the introduction of a scene dataset or an object-location lookup rule. Combining image appearance with 3D structure is useful when the modalities provide different evidence, but disagreement between them must be resolved rather than hidden by an undifferentiated fused representation.

Commonsense also affects navigation preferences in shared spaces. VLM-Social-Nav uses a VLM to score candidate trajectories for socially appropriate robot behavior [15]. This broadens the objective beyond shortest-path movement by incorporating contextual human expectations. However, a model’s social preference is not equivalent to a collision constraint, and social acceptability can vary with users and settings. The relevance of such work lies in adding a contextual evaluation signal to navigation, with its geometric checks and user-study conditions remaining essential to interpretation.

These examples reveal two distinct contributions of prior knowledge. A semantic map helps locate a described entity using accumulated observations; a search prior helps choose which unobserved region to inspect. Conflating them encourages a robot to treat likely object–room associations as confirmed locations. For example, a model may reasonably suggest looking for a cup near a kitchen counter, but an observed cup elsewhere should override that expectation. Similarly, the absence of a target in one image does not establish its absence from a room when occlusion or limited field of view prevents inspection. Grounding must retain the difference between a conjecture, a positive observation, and a genuinely informative negative observation.

The main limitations concern atypical layouts, changes after mapping, visually ambiguous objects, and uncertain spatial relations. A semantic map can be linguistically expressive yet geometrically stale; a correct object category can still identify the wrong instance. A practical research direction is therefore to attach observation provenance and update time to semantic memory, and to reacquire evidence when a task depends on uncertain relations or moved objects. These are proposed requirements for stronger deployment, not capabilities automatically supplied by a pretrained VLM. Evaluation should distinguish target recognition, relation grounding, exploration efficiency, and eventual navigation success, and should test layouts that conflict with common priors. This would reveal whether a method uses knowledge to guide evidence acquisition or merely follows familiar associations.

### 5.3. Decision Correction

**Figure 11:**
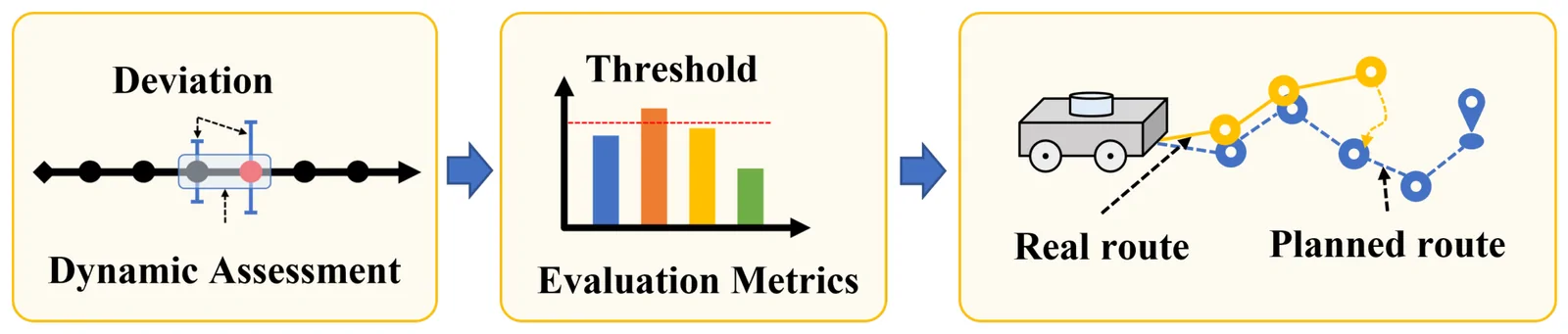
Scheme of decision correction. From left to right, it shows the sequence of the deviation assessment process, the threshold judgment, and the path correction. The black dots represent the sampling status and the red, dashed horizontal lines indicate the assessment threshold. In the path comparison, the blue dashed lines represent the planned trajectory, and the yellow solid lines represent the actual trajectory. The circular marks represent the waypoints. The black dashed arrows highlight the path deviation.

Large models exhibit strong semantic reasoning capabilities, but their outputs are not inherently safe or physically executable. Because most large models were not originally trained for embodied navigation, their predictions may overlook physical constraints or contain hallucinated information. In real-world environments, these limitations are further compounded by perceptual noise and execution uncertainty. If a robot follows a foundation-model-generated plan in an open-loop manner, even minor deviations can accumulate over time and ultimately lead to task failure. A useful correction architecture, as shown in Figure 11, therefore connects the type of observed failure to the module capable of addressing it.

CoNVOI illustrates how contextual decisions can be grounded before execution [133]. It correlates RGB observations with a local occupancy map, marks obstacle-free image locations with identifiers, and asks a VLM to choose a sequence consistent with a contextual behavior prompt. The selected locations form a reference path for a motion planner. When context remains unchanged, the method extrapolates the reference to reduce unnecessary VLM queries. This is a concrete combination of semantic guidance, restricted candidate locations, and lower-level planning, supported by real indoor and outdoor experiments. Restricting choices to marked locations improves the decision interface, but does not establish a universal safety guarantee under mapping error, occlusion, or changed obstacles.

Feedback can also update the task representation. SayNav uses newly observed scene-graph information in dynamic planning [34], while RoboGPT includes replanning alongside its task and skill modules [29]. These mechanisms support revision of semantic subgoals when execution reveals that the original plan is unsuitable. Their significance is the return of embodied evidence to the planner, rather than an assumption that verbal self-correction is sufficient. In a hybrid system, the feedback should identify which goal failed and what changed in the observed environment; an unstructured message that the task failed gives the model little basis for deciding between re-observation, a new route, and a different destination.

Safe-VLN offers a useful execution-level comparison [22]. It filters predicted waypoints using an occupancy mask derived from simulated 2D LiDAR and applies reselection after collision to avoid repeated entrapment. The method is evaluated on R2R-CE and is a task-trained VLN system, not an LLM or VLA architecture. It is relevant here because it demonstrates that collision handling can be supplied by a separate navigation mechanism. Its reduced collisions in the tested simulator do not prove safety for arbitrary physical robots, but the intervention is more specific and verifiable than asking a model whether its own output appears safe.

Decision correction is undoubtedly crucial for large model-based navigation. The most significant aspects among them are timing and memory. Timing is part of this feedback problem. Sensing, perception, model inference, communication, feasibility checking, planning, and actuation all contribute to response delay. If observations become obsolete while a model is reasoning, even a semantically correct decision can be unsuitable at execution time. FASTNav shows one route toward local deployment through compact adapted models [27], while CoNVOI reduces repeated high-level queries when context is stable [133]. These approaches address different costs. A reported language-model response time should not be equated with the controller update period or a complete sensing-to-motion delay. Tail latency, missed deadlines, and the behavior during unavailable model responses are especially relevant in crowded indoor environments. Moreover, memory helps maintain consistency across this loop, but it can also preserve errors. A navigation system should distinguish the intended task, verified scene observations, attempted subgoals, and execution outcomes. Reusing a previous model-generated description as if it were an independent observation can reinforce a hallucinated landmark. The model’s verbal confidence is also not a substitute for sensor uncertainty or measured clearance. The key research problem is thus not merely to accumulate more history, but to determine which parts remain relevant and trustworthy after the environment changes. Therefore, effective decision correction in large model-based navigation requires a balance between timely feedback and reliable memory management, ensuring that navigation decisions remain responsive to environmental changes and grounded in trustworthy observations.

### 5.4. Summary

The predominant role of large model-based navigation in task interpretation and semantic reasoning renders it an inadequate substitute for continuous motion generation. The reviewed studies utilize structured subgoal decomposition, knowledge-guided inference, and feedback-based plan revision to establish a connection between natural language goals and observations as well as executable actions. Their technical contribution lies in making task context and prior knowledge available to navigation decisions that would otherwise be defined mainly in geometric terms. The applicability of these methods, however, is contingent upon the establishment of a solid foundation. The proposed objectives must align with observable entities, accessible locations, and robot actions that are within the feasible range of the robot. Incomplete perception, unverified intermediate reasoning, and slow high-level inference have the potential to disrupt this connection. Consequently, effective systems require interfaces that pass constrained subgoals to lower-level planners, verify critical decisions before execution, and revise plans when feedback indicates a mismatch. The primary outstanding issue is thus the reliability of the reasoning-to-execution interface under physical operating constraints.

## 6. Conclusions and Future Directions

This review has systematically examined the methodological evolution of indoor navigation for autonomous mobile robots (AMRs), from path planning-based navigation to learning-based navigation and, more recently, large model-based navigation. Taken together, the reviewed studies reveal a clear shift in the dominant mechanism underlying navigation decision-making. Classical navigation is primarily built on explicit rules, cost functions, heuristic search, and optimization objectives; learning-based methods extend robot behavior beyond manually designed strategies through data-driven policy learning; and large models further introduce semantic reasoning, task decomposition, commonsense knowledge, and multimodal decision-making into the navigation process.

To provide a more comprehensive assessment of the three navigation paradigms, Table 4 summarizes quantitative performance evidence from different methodological approaches and experimental settings. The results reveal substantial performance variations not only across navigation paradigms but also among methods within the same paradigm. Path planning-based approaches exhibit reliable path generation in structured environments, although their computational efficiency and feasibility depend on environmental complexity and optimization mechanisms. Learning-based methods demonstrate navigation capabilities in previously unseen scenes and physical environments, but their success rates and collision performance vary considerably with task difficulty, training strategies, and environmental conditions. Large model-based methods further enable semantic navigation and instruction-driven decision-making, while their computational requirements and execution efficiency remain dependent on model architectures and inference strategies. Importantly, the heterogeneity of experimental protocols prevents direct numerical ranking across paradigms. Nevertheless, these representative results provide empirical evidence of their distinct performance characteristics and reinforce the need for hybrid navigation architectures that integrate complementary capabilities.

Within this evolutionary trajectory, path planning-based navigation remains highly interpretable, reproducible, and reliable when accurate maps and relatively stable operating conditions are available. Its integration of SLAM, global planning, local obstacle avoidance, and motion control continues to provide a fundamental engineering basis for indoor AMR navigation. Learning-based navigation further enhances the ability of robots to process high-dimensional observations and adapt their behavior to dynamic or partially observable environments. However, its effectiveness remains strongly influenced by reward design, training experience, representation quality, overfitting, and the Sim-to-Real Gap. Building on these capabilities, large model-based navigation extends the objective of navigation beyond geometric goal reaching toward instruction understanding, semantic goal interpretation, long-horizon task decomposition, commonsense reasoning, and closed-loop decision refinement. Navigation-oriented VLA models extend this trajectory toward goal-conditioned action generation, through either hierarchical execution or learned action heads [35,134,135]. Their contribution depends on the action representation and the executor beneath it. Hallucination, grounding error, embodiment mismatch, inference latency, and limited safety verifiability continue to constrain reliable deployment. The central finding of this review is therefore not that one navigation paradigm will ultimately replace the others, but that the development of indoor AMR navigation reflects a progressive enrichment of decision-making capability. The field has evolved from rule-driven execution, to data-driven adaptation, and further toward cognition-driven autonomy, with each paradigm contributing distinct strengths that remain valuable within increasingly integrated navigation systems.

Due to the respective advantages and disadvantages of the three types of methods, the hybrid navigation system has become extremely important in current research. In existing systems, learning-based methods can enhance conventional planners through learned environmental representations, adaptive optimization, or policy-guided motion generation, while geometric planning maintains motion feasibility. Similarly, large models can interpret natural language instructions and generate semantic subgoals, which are subsequently executed through conventional planners or learned navigation policies. Such integration combines the interpretability and reliability of traditional planning, the adaptability of learning-based methods, and the semantic reasoning capabilities of large models. Nevertheless, hybrid systems also introduce challenges related to inter-module coordination, error propagation, and consistency between high-level decisions and low-level execution. These characteristics indicate that the proposed three-paradigm taxonomy should be understood as a classification of dominant decision-making mechanisms rather than a rigid separation of navigation systems, thereby providing a unified perspective for analyzing both individual approaches and their integration.

Several future research directions emerge directly from the limitations identified throughout the reviewed literature. These directions are research recommendations synthesized from the reviewed evidence, rather than capabilities established for all current systems.

- **Lifelong adaptation for long-term tasks**: Existing systems are often trained, mapped, or calibrated for specific environments, whereas real-world deployments involve continual changes in scene configuration, traversability, visual conditions, and task requirements. Future work should therefore develop mechanisms that allow AMRs to update their environmental representations and navigation policies through continued interaction without requiring extensive retraining. A promising strategy is to combine incremental updates of geometric and semantic representations with online memory and execution feedback, allowing environment-specific experience to refine previously learned priors while stable localization, planning, and safety modules maintain reliable operation. Accordingly, evaluation should extend beyond isolated navigation episodes and instead examine whether a system can adapt across sequences of changing indoor conditions without degrading previously acquired capabilities.
- **Simulation with real-world factors**: The gap between simulation and physical deployment also requires closer integration between algorithm development and real-world validation. As shown throughout this review, strong simulation performance does not necessarily translate into reliable navigation on physical robots because sensing noise, execution uncertainty, timing constraints, and environmental variation introduce discrepancies that are difficult to reproduce in simplified settings. Future studies should therefore evaluate navigation systems across both simulated and physical indoor environments under consistent task definitions and comparable performance criteria. Modular system design can further support this process by helping identify whether transfer failures originate from perception, representation, policy learning, planning, or control. In parallel, closed-loop testing can reveal how errors propagate during execution and whether recovery mechanisms remain effective under real operating conditions. Standardized evaluation should consequently include changed layouts, ambiguous instructions, noisy observations, dynamic obstacles, and failure recovery. Success rate (SR) and success weighted by path length (SPL) provide complementary measures of completion and route efficiency [146], but should be accompanied by collision incidence, intervention frequency, recovery time, and timing information. Reports should disclose scene splits, success thresholds, sensing, environmental tours or maps, robot embodiment, and training or fine-tuning data so that within-task comparisons can be interpreted fairly.
- **Integration of embodied memory and world models**: Embodied memory can organize information accumulated during navigation, such as previous observations, semantic relationships, subgoals, and execution outcomes, enabling the robot to refine general priors according to environment-specific experience. World models can complement this capability by predicting plausible state transitions under candidate actions, thereby providing an internal basis for evaluating future navigation consequences before physical execution. Combining such predictive mechanisms with learning-based policies and large model reasoning may improve long-horizon consistency while retaining conventional planning and safety constraints for reliable motion execution. Their effectiveness should be assessed through extended closed-loop navigation tasks, with particular attention to whether accumulated experience and predictive reasoning reduce repeated failures and improve adaptation to changing environments.
- **Real-world indoor AMR deployment**: Deploying large model-based navigation systems on physical indoor AMRs requires addressing environmental uncertainty and system-level constraints. Sensor noise, occlusions, and incomplete observations may compromise perception accuracy and semantic grounding, while communication delays and limited onboard computational resources can further affect real-time decision-making. Beyond these challenges, reliable deployment requires effective coordination between high-level semantic reasoning and safety-critical motion control. Hierarchical navigation architectures offer a promising solution by assigning task interpretation and subgoal generation to large models, while conventional local planners and motion controllers ensure real-time obstacle avoidance and motion safety. Furthermore, execution monitoring and failure recovery mechanisms are essential for detecting infeasible decisions and triggering replanning or safe fallback behaviors under unexpected conditions. Consequently, achieving reliable real-world deployment of large model-based navigation systems remains a significant challenge requiring coordinated advances in perception robustness, computational efficiency, hierarchical decision-making, and safety-aware control.

In conclusion, the evolution of indoor AMR navigation is better understood through complementary decision-making mechanisms than through a ranking of isolated approaches. Progress toward dependable autonomy will require system-level integration of spatial planning, learned adaptation, and semantic reasoning, together with comparable evaluation across task settings and real-world constraints. Such an evidence-oriented perspective can guide the development of navigation systems that are both capable and practically verifiable.

## Figures and Tables

**Table 1:** Representative navigation mechanisms described using common fields. P: path planning-based method; L: learning-based method; F: large model-based method; R: physical indoor ground-robot evidence; S: indoor navigation simulation; A: adjacent-domain or component evidence. Labels describe the stated module and setting, not a mutually exclusive system category.

Method/Year	Task and Inputs	Decision Mechanism and Role	Validation Setting/Evidence	Main Limitation or Evidence Boundary
MEG-RRT* Wu et al., 2026 [16]	Warehouse route planning, known obstacle geometry and start/goal	P: NSGA-II-guided sampling, geometric steering, and shortcuts	MATLAB R2025a narrow-passage and warehouse maps, 100 trials per scenario (S)	Planner feasibility is not physical navigation success, guided samples add CPU overhead.
AHPF-IRL Rousseas et al., 2022 [17]	Point-to-point motion, partially known workspace	P+L: harmonic potential structure with integral RL optimization	Synthetic multiply connected workspace (A)	Analytical properties depend on the formal workspace/control assumptions.
NavACL Morad et al., 2021 [18]	Visual target navigation, RGB observations	L: curriculum-selected tasks, frozen representations, and RL	Simulation, real ground robot and quadrotor (R+S, aerial tests A)	Embodiment and collision conditions must be separated, no general transfer guarantee.
Modular image-goal Wu et al., 2022 [19]	Image-goal navigation, observations, goal image, obstacle map	L+P: learned long-term goals and stopping with reactive motion planning	Simulator and physical mobile-robot scenes (R+S)	Mapping, goal prediction, and stopping errors can propagate across modules.
ANFIS strategy Stavrinidis and Zacharia, 2024 [20]	Goal reaching/obstacle avoidance, simulated ultrasonic sensing	L+P: learned neuro-fuzzy tracking/avoidance rules with explicit switching	Pioneer p_3dx model in CoppeliaSim, static/dynamic obstacles (S)	Controller comparison is simulation evidence, transfer and probabilistic safety are unestablished.
Hybrid depth–VT&R Al-Saedi et al., 2026 [21]	Visual teach-and-repeat, event-derived depth sequences	L: attention-based depth estimation and CNN–LSTM steering	DENSE/MVSEC component evaluation and route scenarios (A)	Path-selection accuracy is not navigation SR, no verified physical indoor AMR deployment.
Safe-VLN Yue et al., 2024 [22]	VLN-CE, instructions, panoramic RGB-D, occupancy mask	L+P: task-trained waypoint/navigation model with occupancy filtering and reselection	Habitat/R2R-CE, simulated pose and LiDAR occupancy (S)	Collision mitigation is not a formal guarantee, sensing and action assumptions limit transfer.
iPPD Wang et al., 2025 [23]	Instruction-guided paths, pre-explored 3D semantic map	P+L: path proposals and learned cross-modal path discrimination	Pre-exploration VLN-CE setting, Matterport3D/Habitat assets (S)	Reported core is map-based proposal/scoring, not an LLM motion policy, pre-exploration changes the protocol.
ETPNav An et al., 2025 [24]	Continuous-space VLN, language and visual observations	L+P: trained cross-modal topological decisions with explicit low-level obstacle handling	R2R-CE and RxR-CE simulation (S)	Map/waypoint errors and trial-and-error control remain, Transformer use alone is not F dominance.
TSOR Huang et al., 2025 [25]	VLN/object referring, instruction and object observations	L: temporal/spatial relation learning and turning-back penalty	REVERIE, SOON, and R2R benchmark tasks (S)	Object detection and benchmark action interfaces constrain interpretation, no t generative LLM reasoning.
DILLM-VLN Wang et al., 2025 [26]	Instruction-following VLN, scene/object features and instructions	L+F: RL action policy aided by LLM sub-instruction decomposition	Matterport3D/R2R-style graph navigation (S)	Quality of decomposition and subgoal completion signals affects the learned policy.
FASTNav Chen et al., 2025 [27]	Multi-point navigation, language tasks and map-related goals	F+L+P: adapted small language models, teacher–student iteration, and Nav2	TurtleBot3 simulation, DIABLO in indoor buildings, Orin NX (R+S)	Environment-specific fine-tuning limits zero-shot claims, model delay differs from motion time.
OVL-MAP Wen et al., 2025 [28]	VLN-CE/Robo-VLN, RGB-D and language	L+F: LSeg open-vocabulary mapping, learned waypoints, DD-PPO/learned action modules	Habitat, R2R-CE and Robo-VLN (S)	Foundation assistance is in semantic representation, online mapping is not physical-robot validation.
RoboGPT Chen et al., 2025 [29]	Long-horizon household instructions, observations and task state	F+L: LLM subgoal planning/replanning with learned skills	AI2-THOR/ALFRED-style embodied tasks (A)	Includes interaction/manipulation, task completion is not pure AMR navigation SR.
HAM-Nav Tan et al., 2025 [30]	Hand-drawn-map navigation, map image and visual landmarks	F+P: GPT-4o association and plan parsing with conventional planners	Gazebo, real Jackal user study, indoor/outdoor and ground/legged settings (R+S, mixed)	Aggregate results mix environments, landmark errors and API-dependent stop-and-go operation remain.
OCNav Park et al., 2025 [31]	Object-centric semantic goals, textual graph nodes and instructions	P+F: explicit confidence ranking with sBERT language grounding and static-aware prior	Semantic topological navigation framework, quantitative protocol not used here (A)	Pretrained embedding retrieval must not be described as generative LLM planning.
NavGPT Zhou et al., 2024 [32]	Zero-shot VLN, textual observations, history, and candidates	F: LLM reasoning and sequential candidate selection	R2R graph-navigation evaluation (S)	Textual summaries can omit geometry, reasoning fluency is not task or safety verification.
NavGPT-2 Zhou et al., 2024 [33]	VLN, visual observations and instructions	F+L: frozen-language-model alignment with a navigation policy	VLN benchmark evaluation (S)	Reasoning supervision and policy adaptation must be reported when describing generalization.
SayNav Rajvanshi et al., 2024 [34]	Multi-object search, incrementally built 3D scene graph	F+L: grounded LLM subgoals and pretrained point-goal execution	ProcTHOR-based MultiON simulation (S)	Search priors and scene-graph errors can misdirect the next subgoal.
Mobility VLA Xu et al., 2025 [35]	Multimodal goal navigation, demonstration-tour video and instruction	F+P: VLM goal-frame selection, topological routing, MPC execution	Physical office/home-like navigation and simulated reconstruction (R+S)	Prior tour and graph are required, high-level goal selection and low-level reaching are distinct outcomes.

**Notes:** Methods in the table are sourced from IEEE Xplore, ScienceDirect, Springer Nature and MDPI’s publishing resources, as well as the self-hosted papers of the authors, arXiv, conference proceedings and Crossref metadata. The topic query combines indoor or mobile robot navigation and path planning, reinforcement learning, visual-language navigation, large language models, visual-language models or visual-language-action models. The key research focuses on the literature published between 2021 and 2026.

**Table 2:** Representative platforms, datasets, and robotic systems for indoor navigation evaluation.

Platform/Dataset	Type/Support	Main Tasks	Key Characteristics	Typical Limitations
Habitat [81]	Embodied AI simulation	PointNav, ObjectNav, VLN	High-throughput rendering of indoor scenes, configurable sensors and task evaluation	Physical fidelity depends on scene assets, sensing, dynamics, and task configuration.
Gazebo [82]	Physics-based robot simulation	SLAM, planning, obstacle avoidance, control	Robot models, physics, rendering, and simulated sensors support system-level testing	Transfer requires calibration of simulated sensors and dynamics, results depend on version and assets.
ProcTHOR [80]	Procedural scenes for AI2-THOR	Visual navigation, generalization evaluation	Large-scale generation of varied indoor layouts supports training and testing	Generated layouts and asset distributions need not match those of a physical deployment site.
Matterport3D [76]	Scanned indoor scene data	Visual navigation, VLN, scene understanding	Aligned RGB-D observations, camera poses, reconstructions, and semantic annotations	Static captures provide no evolving obstacles or layouts, task and action interfaces are defined separately.
Gibson [77]	Scanned scenes with a simulation ecosystem	Visual navigation, embodied learning, transfer studies	Real-building scans support embodied perception with visual and geometric observations	Scan coverage and rendering and physics assumptions bound the diversity and fidelity of evaluation.
R2R [10]	Instructions and routes on Matterport3D	Language-guided navigation	Human instructions are grounded in paths through reconstructed indoor environments	Standard graph-based actions omit continuous motion control and collision avoidance.
REVERIE [79]	Referring expressions and object targets	Navigation and object grounding	Remote target descriptions require both reaching a relevant viewpoint and identifying the object	Standard discrete evaluation combines navigation and grounding, it does not establish safe physical execution.
TurtleBot3 [83]	Compact ROS mobile base	SLAM, planning, indoor navigation trials	Open-source differential-drive platform with modular hardware for research and prototyping	Payload and sensing constraints vary by model, visual-navigation tests require explicit camera configuration.
Jackal [84]	Indoor/outdoor mobile base	Planning, sensor fusion, physical navigation trials	ROS-based ground robot with configurable sensor payloads for real-world testing	Performance depends on payload, platform configuration, and site conditions, indoor and outdoor results must be separated.

**Notes:** PointNav denotes point-goal navigation, ObjectNav denotes object-goal navigation. Capabilities depend on the resource version and configuration. Limitations summarize evaluation boundaries rather than a measured ranking of platform fidelity.

**Table 3:** Representative large model-assisted navigation interfaces and their evidence boundaries.

Method	Model Role and Output	Execution Interface	Evaluation and Interpretation Boundary
NavGPT [32]	LLM reasoning, candidate-viewpoint choice	Discrete navigation graph	Zero-shot R2R, below trained specialists in the original study, no continuous-control validation implied.
NavGPT-2 [33]	Visual alignment with a frozen LLM, navigation policy	Task-trained viewpoint policy	VLN benchmarks, navigation-specific training contributes to performance.
SayNav [34]	LLM subgoals grounded in an incremental scene graph	Pretrained point-goal planner	Unseen-environment multi-object navigation, relies on perception and the local planner.
FASTNav [27]	Compact adapted language model, ordered goals	Nav2	Simulation and real robots, task/map adaptation differs from zero-shot building transfer.
OVL-MAP [28]	Pretrained semantic map features, learned waypoints	DD-PPO action module	Continuous Habitat simulation, semantic pretraining is distinct from LLM planning.
HAM-Nav [30]	VLM interpretation of sketch landmarks	Conventional global/local planners	Simulation and real Jackal study, aggregate indoor/outdoor results.
CoNVOI [133]	VLM contextual selection of marked free-space locations	Reference path and motion planner	Real indoor/outdoor navigation, relies on local occupancy grounding.
Mobility VLA [35]	Tour-conditioned goal-frame selection	Topological routing and MPC	Physical office navigation with an environmental tour prior.
NaVILA [134]	VLA spatial mid-level commands	Learned locomotion policy	Continuous simulation and physical legged robots, embodiment-specific execution.
OmniVLA [135]	Multimodal goal conditioning, action sequences	Learned navigation action head	2025 preprint, mixed indoor/outdoor evaluation, separate large and edge variants.

**Table 4:** Comparisons across indoor AMR navigation paradigms.

Paradigm/Method	Navigation Success	Computational Cost	Online Latency	Dynamic Adaptability	Sim-to-Real/Real-World Validation	Safety
Path Planning-Based Navigation						
CAF-RRT* [85]	Path cost reduced by 2.18% vs. RRT*	NR	Planning time reduced by 97.25% vs. RRT*	Complex static maps	ROS and robot validation	Clearance-aware path smoothing
APSO [86]	Travel time reduced by 17.35% vs. A*	Additional optimization overhead	NR	Static maps	Simulation	Collision-free geometric planning
MEG-RRT* [16]	Path-search SR: 100%	Evolutionary optimization overhead	Planning: 0.3377 s	Structured static warehouse	Simulation	Collision-free paths; safety-margin constraints
KA-IHO [93]	Path-search SR: 80–100% (selected maps)	NR	NR	Static obstacle-dense maps	Physical-robot validation	Collision-free execution; tracking error within ±4 cm
MOD-RRT* [88]	NR	NR	NR	Dynamic replanning evaluated	Simulation and practical experiments	Obstacle avoidance demonstrated
Learning-Based Navigation						
End-to-end Visual DRL [59]	Real-world SR: 86.7%	Training: approx. 30 GPU-h	NR	NR	Real-world deployment after adaptation	NR
TD-RL-ITR [66]	Unseen SR: 44%; SPL: 0.22	NR	NR	Crowded-scene evaluation	Physical tests in [19]	Real-world CR: 38.8%
NavACL [18]	Unseen SR: 42%; SPL: 0.24	NR	NR	Unseen cluttered scenes	Transfer without policy retraining	NR
Modular Image-Goal [19]	Unseen SR: 57%; SPL: 0.35	NR	NR	Complex real-world scenes	Simulation-trained modules deployed on real robot	Real-world CR: 16.0%
Spatiotemporal Attention [113]	NR	Lightweight LiDAR-based controller	NR	Moving-obstacle navigation evaluated	Real-robot deployment	Collision avoidance evaluated qualitatively
Large Model-Based Navigation						
DILLM-VLN [26]	Unseen SR: 49.4%; SPL: 0.44	NR	NR	NR	Simulation evaluation	NR
FASTNav [27]	Task SR: 70.0%	Model memory: 8 GB	Inference: 2.87 s	Mostly stable landmarks	Simulation and real-robot testing	Low-level control delegated to Nav2
HAM-Nav [30]	Simulation SR: 80%; SPL: 0.712	NR	Navigation duration: 634 s	NR for moving obstacles	Real-world SR: 78%; SPL: 0.714	Conventional local planning
NavGPT-2 [33]	Unseen SR: 73.82%; SPL: 61.06%	NR	NR	NR	R2R simulation benchmark	NR
SayNav [34]	SR improvement >8% vs. oracle baseline	NR	NR	Online semantic replanning	ProcTHOR simulation	Pretrained low-level navigation policy

**Notes:** The RRT* and A* referenced in the table are names of methods. The asterisk does not indicate a special explanation. SR: success rate; SPL: success weighted by path length; CR: collision rate; NR: not reported or not reliably established. The results are obtained from studies employing different navigation tasks, experimental environments, robotic platforms, and computational configurations. Path-search success rates should not be interpreted as physical navigation success rates. Planning time, inference latency, training cost, and total navigation duration represent distinct measurements. Localization accuracy is often determined by an independent SLAM subsystem and is not necessarily attributable to the navigation decision-making paradigm. Adaptation to unseen static scenes does not necessarily imply robustness to moving obstacles. Real-world validation does not always demonstrate direct sim-to-real transfer without retraining. The reported results therefore provide representative experimental evidence rather than directly comparable cross-paradigm rankings.

## Data Availability

No new experimental data were generated in this study. The comparative analyses are based on the previously published literature. The quantitative results summarized in this review are provided in the article, with references to the original sources.

## Abbreviations

The following abbreviations are used in this manuscript:

AMR	Autonomous mobile robot
DRL	Deep reinforcement learning
SLAM	Simultaneous localization and mapping
LLM	Large language model
VLM	Vision–language model
VLA	Vision–language–action
VLN	Vision–language navigation
CoT	Chain-of-thought
MPC	Model predictive control
SR	Success rate
SPL	Success weighted by path length
